# Sanggenol L Induces Endoplasmic Reticulum Stress and Triggers Cell Apoptosis in Glioblastoma by Binding to BiP

**DOI:** 10.7150/ijbs.126892

**Published:** 2026-04-16

**Authors:** Jingyang Xu, Hongbo Chang, Yi Du, Zihan Tang, Ziyang Qin, Yongzhao Wang, Fujing Wei, Xiaosong Hu, Jianbing Hou, Man Xu, Hongjuan Cui

**Affiliations:** 1 State Key Laboratory of Resource Insects, Medical Research Institute, Southwest University, Chongqing 400715, China.; 2 Jinfeng Laboratory, Chongqing 401329, China.

**Keywords:** Sanggenol L, Glioblastoma, BiP, Chemosensitivity

## Abstract

*Morus alba L.* (white mulberry) is a medicinal plant whose extracts possess significant anti-tumor potential. Through screening 59 commercially available mulberry-derived compounds, we identified a small molecule, Sanggenol L (SL), which exhibited potent inhibitory effects on glioblastoma while showing minimal toxicity toward normal cells. This study aimed to elucidate the anti-cancer function and underlying mechanism of SL in glioblastoma cells. Experimental results demonstrated that SL induced cytotoxic endoplasmic reticulum (ER) stress, suppressed cell viability, and triggered apoptosis. Mechanistically, LC-MS/MS and CETSA analyses revealed that SL specifically binds to BiP, creating a steric hindrance that blocks its interaction with IRE1α. This leads to excessive dimerization of IRE1α and hyper-activation of the IRE1/CHOP/XBP1s/Bcl-2 pathway, thereby amplifying cytotoxic ER stress and ultimately promoting apoptosis. Furthermore, we found that SL promotes ubiquitination and degradation of MGMT, consequently enhancing the chemosensitivity of glioblastoma to temozolomide.

## Introduction

Glioblastoma (GBM) is the most aggressive and lethal form of primary brain tumor, characterized by rapid progression, diffuse infiltration and a high degree of cellular heterogeneity 1. Despite advancements in surgical techniques, radiation therapy and chemotherapy, the prognosis for GBM patients remains only about one year after diagnosis 2. The standard management of GBM involves maximal safe resection, followed by concurrent and adjuvant chemotherapy with temozolomide (TMZ). A primary cause of therapeutic failure is the overexpression of O^6^-methylguanine-DNA methyltransferase (MGMT). This enzyme confers resistance by enzymatically reversing the DNA alkylation damage caused by TMZ 3. Furthermore, the complexity of the GBM tumor microenvironment, which includes cancer stem cells, immune evasion mechanisms, and active angiogenesis, adds a major layer of therapeutic difficulty. This underscores the urgent need to develop novel agents capable of overcoming TMZ resistance and directly targeting core GBM vulnerabilities.

Natural products have consistently served as important sources of anticancer agents. Notably, *Morus alba L.* (mulberry), which holds a prominent place in traditional Chinese medicine, contains numerous bioactive compounds with well-documented pharmacological activities 4. It is renowned for a wide range of therapeutic benefits, including antioxidant, anti-atherosclerotic, blood glucose-regulating, immunomodulatory, and neuroprotective effects 5. The therapeutic efficacy is primarily attributed to bioactive constituents, such as flavonoids, anthocyanins, and phenolic acids, among others 6.

Sanggenol L (SL, CAS No. 329319-20-2), a natural compound derived from the root bark of *Morus alba L.*, is the focus of this study and has attracted significant interest in pharmaceutical research for its potential in cancer therapy. SL has been reported to exert anticancer effects in multiple models. For instance, it promotes apoptosis in melanoma cells by activating the caspase cascade and apoptosis-inducing factor, while in ovarian cancer cells, it acts through caspase activation and subsequent inhibition of the NF-κB pathway. Furthermore, in human prostate cancer cells, SL induces both apoptosis and cell cycle arrest by activating p53 and suppressing the PI3K/Akt/mTOR pathway 7-9. While SL has demonstrated significant effects on multiple signaling pathways and antitumor potential, its direct target remained unknown. To address this, we employed LC-MS to identify the target protein of SL. Our study reveals its role in inducing endoplasmic reticulum (ER) stress in GBM cells. Although other bioactive compounds from mulberry have been suggested to influence ER stress, their specific targets and mechanisms of action are largely unclear. In contrast, our work not only identifies and validates the direct target of SL, clarifying how it triggers ER stress, but also uncovers its significant potential in chemotherapy sensitization.

This study investigated the anticancer effects of SL on GBM. We found that SL binds to BiP, disrupting its interaction with IRE1α. This results in unrestrained IRE1α dimerization, which activates the CHOP/XBP1s/Bcl2 axis and amplifies ER-stress-mediated apoptosis 10. We also demonstrated that SL enhances TMZ chemosensitivity in GBM, mechanistically linked to its promotion of MGMT ubiquitination and degradation. A summary of this mechanism is presented in Figure 8. These results collectively elucidate the pharmacological profile of SL, which may pave the way for new targeted therapeutic strategies against glioblastoma.

## Materials and Methods

### Regents and antibodies

Sanggenon L (Cat#DS0100) was procured from LEMEITIAN MEDICINE (Chengdu, China) and dissolved in DMSO. Temozolomide (Cat#HY-17364) was obtained from MedChemExpress (New Jersey, USA). The Glutamic oxalacetic transaminase/Aspartate aminotransferase (GOT/AST) Kit (Cat#G0424W), Glutamic pyruvic transaminase/Alanine aminotransferase (GPT/ALT) Kit (Cat# G0423W), Albumin (ALB) content Kit (Cat#G1208W) and Protein Content (SP) Kit (Cat#G0432W) was obtained from Grace Biotechnology (Suzhou, China). The antibodies anti-BiP (Cat#11587-1-AP), anti-IRE1 (Cat#27528-1-AP), anti-XBP1S (Cat#83959-5-RR), anti-CHOP (Cat#15204-1-AP), anti-MGMT (Cat#17195-1-AP), anti-CDK1 (Cat#19532-1-AP), anti-Cyclin B1 (Cat#55004-1-AP), anti-Alpha Tubulin (Cat#80762-1-RR), anti-MYC tag (Cat#60003-2-Ig), anti-DYKDDDDK tag (Cat#20543-1-AP), and anti-HA tag (Cat#51064-2-AP) were sourced from Proteintech (Wuhan, China). Additional antibodies, including anti-Cleaved Caspase-3 (Cat#9661T), anti-Cleaved Caspase-7 (Cat#9491T), anti-Cleaved Caspase-9 (Cat#9509T), anti-Caspase-12 (Cat#58208T), and anti-Bcl-2 (Cat#15071T), were acquired from Cell Signaling Technology (Boston, MA, USA). MTT (Cat#M5655), MG132 (Cat#M7449), and DMSO (Cat#D5879) reagents were purchased from Sigma-Aldrich (St. Louis, MO, USA). Reagents such as DAPI (Cat#C1002), the BeyoClick™ EdU Cell Proliferation Kit with Alexa Fluor 488 (Cat#C0071S), the Fluo-4 Calcium Assay Kit (Cat#S1061S), the Comet Assay Kit (Cat#C2041S), the BCA Protein Assay Kit (Cat#P0012), the RIPA Lysis Buffer (Cat#P0013B), the Cell Lysis Buffer for Western and IP (Cat#P0013), the Crystal Violet Staining Solution (Cat#C0121), HRP goat anti-mouse antibody (Cat#A0126), HRP goat anti-rabbit antibody (Cat#A0208) were sourced from Beyotime (Shanghai, China). The Blue/Clear Native PAGE Electrophoresis Kit (Cat#RTD6140) was acquired from Real Times (Beijing, China). Finally, the Goat anti-Rabbit IgG (H+L) Cross-Adsorbed Secondary Antibody, Alexa Fluor™ 488 (Cat#A-11008), the Goat anti-Rabbit IgG (H+L) Cross-Adsorbed Secondary Antibody, Alexa Fluor™ 594 (Cat#A-11012) and the transfection reagent Lipofectamine™ 2000 was purchased from Thermo Fisher Scientific (New York, USA).

### Cell culture

GBM cell lines (LN-229 and T98G), normal astroglia cells (SVGP12), and human embryonic kidney cells (HEK293T and HEK293FT) were purchased from the American Type Culture Collection (ATCC, USA). All cell lines were confirmed to be mycoplasma-free and were cultured as previously described. Temozolomide (TMZ)-resistant cell lines were developed from GBM-3^11^ and T98 G by gradually increasing the TMZ concentration in the culture medium from 10 μM to 500 μM (increments of 10, 20, 40, 80, 120, 160, 200, 250, 300, 350, 400, and 500 μM).

### Colony formation assay

Using a colony formation assay, the impact of Sanggenol L on the clonogenic potential of GBM cells was assessed. Cells (1000 per well) were seeded into six-well plates. Following attachment, the cells were treated with SL at the indicated concentrations. After approximately two weeks of incubation, colonies were fixed, stained with crystal violet, and enumerated in each well using ImageJ software.

### Cell proliferation analysis

Cell viability was determined by MTT assay. Briefly, cells were seeded in 96-well plates at a density of 2000 cells per well (in triplicate) and allowed to adhere overnight. Subsequently, the cells were treated with complete medium containing the indicated concentrations of the SL. At the indicated time points, 20 μl of MTT solution was added to each well, followed by incubation for 3 hours. The culture medium was then removed, and 200 μl of DMSO was added to dissolve the formazan crystals. Absorbance, reflecting both cell number and viability, was measured using a microplate reader at 560 nm.

### EdU staining

Cell proliferation was assessed using EdU staining according to the manufacturer's instructions. Briefly, 2×10⁴ cells were seeded per well in 24-well plates and cultured overnight. The cells were then treated with SL or DMSO for 48 hours. Following treatment, the cells were incubated with 10 μM EdU for 2 hours, fixed with 4% paraformaldehyde for 15 minutes, and permeabilized with 0.3% Triton X-100 for 10 minutes. A Click reaction cocktail was then applied for 30 minutes. Finally, nuclei were counterstained with DAPI for 30 minutes at room temperature, and the samples were examined under a microscope.

### Flow cytometry

GBM cells were treated with SL or DMSO for 48 hours, then harvested by digestion and centrifugation. The cell pellets were subsequently resuspended in PBS buffer. For cell cycle analysis, cells were fixed in 75% ethanol for at least 24 hours, stained with propidium iodide (PI) and RNase, and then analyzed by flow cytometry. All experiments were performed in triplicate. For apoptosis detection, cells were stained with FITC-conjugated Annexin V and PI for 30 minutes and analyzed via flow cytometry using an Annexin V-FITC/PI Apoptosis Detection Kit (Beyotime, Shanghai).

### Transfection and infection

A short hairpin (sh) RNA targeting IRE1 was cloned into the pLKO.1-puro plasmid (shRNA sequences are provided in Supplementary Table S4). Flag-tagged IRE1 and MYC-tagged BiP plasmids were obtained from Yubio (Wuhan, China). For transfection, the indicated plasmids were introduced into HEK 293FT cells using Lipofectamine 2000 (Thermo Fisher Scientific, USA) according to the manufacturer's protocol. Viral supernatant or cells were harvested 48 h post-transfection. For infection, GBM cells were incubated with the viral supernatant in the presence of polybrene (Sigma-Aldrich, USA). Following two rounds of infection, stable cells were selected and pooled under puromycin (Sigma-Aldrich, USA) treatment.

### Western blot

Cells were harvested with a cell scraper and washed three times with PBS. Subsequently, the cells were lysed on ice using RIPA lysis buffer supplemented with 1 mM protease and phosphatase inhibitor cocktail. Protein concentration was measured with a BCA assay kit. Then, 40-60 µg of protein was mixed with 5× loading buffer and heated at 100°C for 15 min. The samples were separated by SDS-PAGE and transferred onto a PVDF membrane (Millipore, Germany). After blocking with 5% BSA for 2 h at room temperature, the membrane was incubated with primary antibody, followed by an HRP-conjugated secondary antibody. When performing Co-IP, detection should be carried out with a primary antibody that is species-mismatched to the immunoprecipitating antibody to circumvent issues related to heavy/light chain interference. Protein signals were finally detected using an ECL detection system (Clinx, Shanghai).

### Label free analysis

Following a 48-hour treatment with SL or DMSO, LN-229 or T98G cells were collected and subjected to label-free quantitative proteomic analysis (performed by Shanghai Applied Protein Technology Biotechnology Co., Ltd). The experimental workflow included protein extraction, enzymatic digestion of peptides, liquid chromatography-tandem mass spectrometry (LC-MS/MS) data acquisition, and database searching. Protein expression differences were assessed by comparing the numbers of upregulated and downregulated proteins between groups. A protein was considered significantly upregulated if its fold change (FC) was > 2.0, or downregulated if FC was < 0.5, with a p-value < 0.05 set as the threshold for statistical significance.

### Ubiquitination assay and protein turnover assay

The designated plasmids were co-transfected into HEK 293T cells for the ubiquitination assay, following a previously published protocol 12. Following plasmid transfection for 36 hours, cells were treated with either SL at the indicated concentration or DMSO (control) for an additional 36 hours. MG132 was added 8 hours prior to cell harvest to inhibit proteasomal degradation. Cells were then lysed on ice using IP lysis buffer supplemented with 1 mM protease and phosphatase inhibitors. Co-immunoprecipitation (Co-IP) was subsequently performed to assess the effect of SL on the ubiquitination level of the target protein.

For the protein turnover assay, GBM cells were treated with SL or DMSO, followed by exposure to 100 μg/mL cycloheximide (CHX) for the indicated time intervals. Cells were harvested and analyzed by western blotting.

### Animal studies and animal ethics

The experiments were conducted as previously described. Briefly, thirty-six female NOD/SCID mice, four weeks old (supplied by Slike Jingda Laboratory Animal Co., Ltd., Hunan, China; Animal qualification number: SYXK-2022-0008), were housed in SPF room.

Firstly, an LN-229 cell line stably expressing luciferase was established. NOD/SCID mice were anesthetized via intraperitoneal injection of 0.7% sodium pentobarbital and placed in a prone position with the head fixed on a stereotactic apparatus. After disinfection with 75% ethanol, the skin on the top of the head was incised. A 1 mm diameter animal skull drill was used to penetrate the skull anterior and lateral to the lambda point. Using a micro-syringe loaded with 5-10 μL of LN-229-Luciference cell suspension (1×10⁵ cells), the needle was vertically inserted into the cerebral white matter through the burr hole. The insertion depth was set so that the needle tip reached 3.5 mm below the cranial surface. Before injection, the needle was slightly retracted by approximately 0.5 mm. The tumor cell suspension was then slowly infused at a rate of 0.5 μL/min. The needle was left in place for 2 minutes post-injection before being gradually withdrawn. The burr hole was sealed with bone wax, and the scalp was sutured and disinfected with iodine. After three days, the mice received intravenous injections of SL (30 mg/kg) every two days for a period of 20 days. Control mice were administered DMSO injections. In this study, soybean phosphatidylcholine, cholesterol, DSPE-PEG2k-Biotin, DSPE-PEG2k-HA, and IR-780 were used to encapsulate SL, with SL being loaded within the bilayer to form functionalized liposomal nanoparticles. Based on the physicochemical properties of SL, the ethanol injection method was chosen to prepare the nanoparticles in this study. For detailed procedures, as well as supporting data on drug release and distribution in various organs, have been reported in our previous study 13. Tumor quantification was performed in a randomized and single-blind manner, as previously described. The animal experiments were approved by the Institutional Animal Care and Use Committee of Southwest University (Ethics approval serial number: IACUC-20240109-04) and were conducted in accordance with the Guide for the Care and Use of Laboratory Animals (Ministry of Science and Technology of China, 2006).

### *In vivo* imaging

The *in vivo* imaging experiment was conducted using the IVIS SpectrumCT system, employing BALB/c nude mice as the animal model. Glioblastoma (LN-229) cells, tagged with luciferase, were injected directly into the brains of the mice, and *in vivo* imaging was carried out following tumor formation. Prior to the experiments, the mice underwent a 12-hour fasting period and were subsequently anesthetized via intraperitoneal injection of a 0.7% sodium pentobarbital solution. Following anesthesia, D-luciferin potassium salt was administered as a substrate at a dosage of 150 mg/kg. After a 10-minute interval, the mice were placed in the imaging dark box, and imaging was performed using designated excitation and emission filters. The imaging parameters included a 1-minute exposure time, carefully set to ensure signal stability. Image analysis was conducted using the Living Image software, which was utilized to calculate both the luminescence area and the total photon count 13. To monitor the progression of the orthotopically implanted tumors within the brain, *in vivo* imaging was conducted on days 7, 15, and 21 post-intracranial injection.

### Subcutaneous transplantation

A subcutaneous transplantation assay was performed using GBM 3 cells. Following proper anesthesia and disinfection, one million cells were injected into the dorsal subcutaneous tissue of BALB/c nude mice (SPF grade, with an average weight of 16-18 g). Tumor size was monitored every two to three days. Upon initial tumor formation, the mice received intraperitoneal administration of DMSO, SL (30 mg/kg), TMZ (30 mg/kg), or a combination of SL and TMZ. Treatments were administered every other day. Prior to tumor collection, the mice were anesthetized by intraperitoneal injection of 0.7% sodium pentobarbital to minimize discomfort. Tumor dimensions were measured and photographed for documentation.

### Fluo-4 calcium assay kit detection

Intracellular calcium ion levels were measured using the Fluo-4 AM fluorescent probe. Briefly, 2×10⁴ cells were seeded per well of a 24-well plate and allowed to adhere overnight. After 48-hour treatment with SL, the culture medium was removed and the cells were washed with PBS. An appropriate volume of Fluo-4 AM staining solution was then added, and the cells were incubated for 30 minutes. Fluorescence images were acquired using a fluorescence microscope with an emission wavelength of 525 nm.

### Native-PAGE

Blue/Clear Native PAGE (BN-PAGE, Real Times, Beijing) is an electrophoretic technique for separating native protein complexes from biological samples. In this method, Coomassie Brilliant Blue G-250 (instead of SDS) binds to proteins and confers a negative charge, enabling separation in a polyacrylamide gel based on molecular weight. GBM cells treated with SL or DMSO for 48 h were harvested using a cell scraper and lysed on ice for one hour in IP cell lysis buffer. The supernatant was then mixed with an appropriate amount of 4× BN/CN-PAGE protein loading buffer and 5% G-250 staining solution (for loading). Electrophoresis was performed using a Native PAGE gel system. Following electrophoresis and transfer, the PVDF membrane (Millipore, Germany) was blocked with 5% BSA for two hours at room temperature. Target proteins were detected by incubation with HRP-conjugated primary and secondary antibodies, and bands were visualized using an ECL detection system (Clinx, Shanghai).

### Cell thermal shift assay (CETSA)

HEK 293T cells overexpressing the target protein were treated with a high concentration of SL for 2-4 hours, followed by trypsin digestion. The cells were then washed once or twice with PBS and resuspended in 400 µL of PBS. Subsequently, the suspension was divided into 7 aliquots of 50 µL each. A suitable temperature range and gradient were selected; in this study, a range of 48 °C to 74 °C was applied. Each aliquot was exposed to the designated temperature for 3-5 minutes, then subjected to repeated freeze-thaw cycles using liquid nitrogen. After high-speed centrifugation (20000 g, 20 min), the supernatant was collected. An appropriate amount of loading buffer was added, and samples were analyzed by Western blot. Quantitative analysis was performed to determine whether the compound enhanced the thermal stability of the target protein.

### Comet assay

The Single Cell Gel Electrophoresis Assay, commonly known as the Comet Assay, was employed to detect DNA damage in individual cells. In this experiment, GBM cells treated with either SL or DMSO for 48 hours were detached using trypsin. Following a standard protocol, a two-layer comet assay slide was prepared: the first layer consisted of 1% normal-melting-point agarose, and the second layer contained approximately 1×10⁴ cells suspended in 0.7% low-melting-point agarose. After lysis, the samples were electrophoresed under specified conditions, neutralized, and stained for visualization of comet tails under a fluorescence microscope, using an emission wavelength of 617 nm.

### Epoxy-activated sepharose 6B affinity media

Epoxy-activated Sepharose 6B affinity medium was first allowed to swell. SL or DMSO was then incubated with the medium overnight at room temperature with gentle agitation. No antibody was involved in this step. After incubation, the medium was washed thoroughly for at least three cycles. Remaining active groups were blocked with 1 M ethanolamine for 4 hours at 37 °C. The SL- or DMSO-coupled medium was subsequently incubated with cell lysate overnight at 4 °C under gentle agitation. Following incubation, the medium was again washed for a minimum of three cycles. An appropriate amount of loading buffer was added, and the samples were heated in a 100 °C water bath for 15 minutes. The resulting supernatant was then collected for SDS-PAGE electrophoresis.

### Hematoxylin-eosin (H&E) staining

The brain tissue of mice was fixed in 4% PFA for about 48 hours. Following fixation, tissue samples were dehydrated through a graded ethanol series (70%, 80%, 90%, and absolute ethanol), with each step lasting 30 to 60 minutes. The dehydrated tissues were then cleared by sequential incubation in a 1:1 xylene-ethanol mixture and pure xylene, each for one hour. Subsequently, the tissues were infiltrated with molten paraffin overnight, embedded in paraffin blocks, and sectioned. The paraffin sections were baked at 65 °C to melt the surrounding wax. Deparaffinization and rehydration were performed by sequentially treating the sections with xylene, a 1:1 xylene-ethanol mixture, absolute ethanol, and a descending ethanol series (90%, 80%, 70%), with each treatment lasting 10 to 20 minutes. For staining, the sections were first stained with hematoxylin, followed by differentiation in HCl and bluing in a weak ammonia solution. After adequate nuclear staining, the cytoplasm was counterstained with eosin. Finally, the sections were dehydrated and cleared by rapid passage through an ascending ethanol series (70%, 80%, 90%, absolute ethanol), a 1:1 xylene-ethanol mixture, and pure xylene, with each step lasting 3 to 5 minutes, before being mounted for microscopic observation.

### Immunohistochemistry assay

The tumor tissue was fixed in 4% paraformaldehyde (PFA) and embedded in paraffin. Sections were cut and baked, then deparaffinized in xylene and rehydrated through a graded ethanol series. Antigen retrieval was performed, followed by blocking of endogenous peroxidase activity. After blocking with 5% goat serum, the sections were incubated with the corresponding primary antibody overnight at 4 °C. Subsequently, a secondary antibody was applied at room temperature, followed by detection with diaminobenzidine (DAB). Nuclei were counterstained with hematoxylin. Finally, the sections were dehydrated, cleared, mounted, and observed under a microscope.

### Immunofluorescence

Immunofluorescence was performed to assess the effect of SL on the interaction between BiP and IRE1α. GBM cells were plated on glass coverslips placed in 24-well plates and treated with 10 μM or 20 μM SL for 48 h. Cells were then fixed with 4% PFA, permeabilized with 0.2% Triton X-100, and blocked with 5% goat serum. Subsequently, cells were incubated with an anti-IRE1α primary antibody for 2 h at room temperature, followed by an appropriate fluorescent secondary antibody. Afterwards, samples were incubated with an anti-BiP primary antibody under the same conditions, followed by a differently labeled fluorescent secondary antibody. Nuclei were counterstained with DAPI, and fluorescence signals were visualized using confocal microscopy.

### Renal and hepatic function tests

Blood samples were collected from mice prior to and 12 hours after drug administration, and then diluted according to the kit instructions (GOT/AST kit, GPT/ALT kit, ALB kit, SP kit), followed by loading the samples accordingly (Grace Biotechnology, Suzhou). The data obtained from the microplate reader were analyzed based on the calculation method provided in the manufacturer's protocol. A comparison was made between the levels of transaminases and proteins in the blood before and after the animals were injected with either SL or DMSO. The effects of the drug on liver and kidney function were evaluated.

### Statistics analysis

All data were presented independently three times (n = 3) and analyzed using GraphPad Prism 8. Results are expressed as Mean ± SD. Statistical significance was assessed for independent samples using the student's unpaired t-test or Two-way ANOVA (or mixed model), with significance defined as p < 0.05 (p < 0.05: *, p < 0.01: **, p < 0.001: ***). Details of the statistical analysis, including the n value, p value, and corresponding t- or F-value, had be provided in the supplementary materials.

## Results

### Sanggenol L was found to significantly suppress the proliferation of glioblastoma cells both *in vitro* and *in vivo*

**Figure 1:**
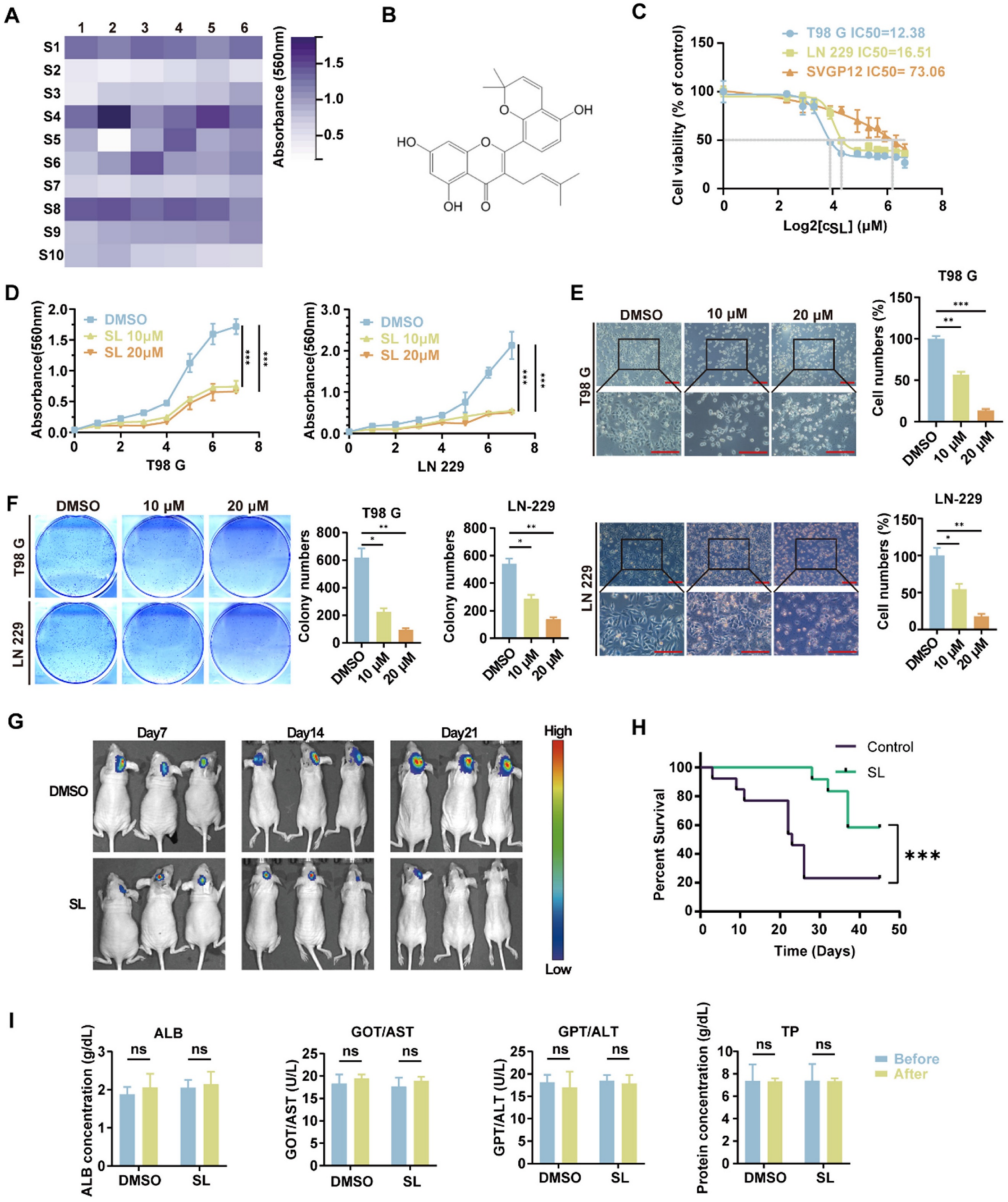
** To detect the effect of Sanggenol L on the proliferation of glioblastoma.** (**A**) A colony formation assay was performed in which glioblastoma cells (LN-229) were treated with 59 active compounds derived from mulberry at a concentration of 15 µM. Cell colonies were stained with crystal violet, washed with PBS to remove background staining, and eluted with absolute ethanol. The absorbance was measured at 560 nm using a microplate reader. Each square in the grid represents a different compound, with the horizontal and vertical axis labels indicating the compound number. The S1-01 (DMSO) group served as the control. (**B**) Chemical structure of Sanggenol L (hereafter referred to as SL). (**C**) The IC_50_ of SL was determined in glioblastoma cells (T98G and LN-229) and normal glial cells (SVGP12) using the MTT assay. Cells were treated with a gradient concentration of SL (0, 5, 7.5, 10, 15, 20, 30, 40, 50, 60, 80, 100 µM) for 48 hours, followed by MTT assay to assess cell viability. The IC_50_ values of SL were 12.38 µM in T98G cells and 16.51 µM in LN-229 cells. (**D**) The proliferation-inhibitory effects of SL at 10 µM and 20 µM were evaluated in glioblastoma cells (T98G and LN-229). Cell viability was measured by MTT assay after 1, 2, 3, 4, 5, 6, and 7 days of treatment. The DMSO group served as the control. (**E**) Morphology and state of glioblastoma cells (T98G and LN-229) were examined under a microscope after treatment with different concentrations of SL. The DMSO group was used as the control. Scale bars = 200 µm. (**F**) The effect of SL on the colony-forming ability of glioblastoma cells (T98G and LN-229) *in vitro* was assessed after treatment with the indicated concentrations. The DMSO group served as the control. (**G**)* In vivo* imaging was performed to evaluate the therapeutic efficacy of SL (30 mg/kg) in mice bearing orthotopic glioblastoma xenografts. SL was encapsulated using soybean phosphatidylcholine, cholesterol, DSPE-PEG2k-Biotin, DSPE-PEG2k-HA, and IR-780, and administered via intravenous injection. (**H**) Survival of mice with orthotopic xenografts was monitored, and a survival curve was generated. (**I**) Blood samples were collected from mice before and 12 hours after SL administration. After dilution according to the kit manufacturer's instructions, transaminase levels, albumin, and total protein content were measured to assess the effects of SL on liver and kidney function. All data is shown as the means ± SD; n=3, *p < 0.05, **p < 0.01, ***p < 0.001.

To identify mulberry-derived active substances with the potential to inhibit glioblastoma (GBM) progression, we first conducted an analysis of 623 mulberry-related compounds using the Traditional Chinese Medicine Systems Pharmacology Database. The compounds were sourced from various parts of the plant: 194 from *Morus alba L.* root bark, 46 from Parasitic Loranthus, 91 from mulberry fruit, 269 from mulberry leaves, and 23 from mulberry twigs. These compounds were evaluated based on key parameters including drug-likeness, number of hydrogen bond donors and acceptors, and lipid-water partition coefficient. Following this preliminary screening, 59 small-molecule active monomers from mulberry extracts were selected for further study based on their potential medicinal value (Supplementary Table S1). Using a colony formation assay, we treated the glioblastoma (GBM) cell line LN-229 with a series of compounds, using DMSO as the control. The results identified several molecules with significant inhibitory effects. The six most active small-molecule compounds were: Albanol B (S5-2), Sanggenol L (S2-2), Sanggenon C (S3-1), Morusin (S2-1), Kuwanon A (S2-3), and 3'-geranyl-3-prenyl-2',4',5,7-tetrahydroxyflavone (S2-6) (Figure 1A, Supplementary Figure S1A). In our team's previous studies, the function and mechanisms of Albanol B in glioblastoma cells have already been demonstrated 14. Therefore, we turned our attention to Sanggenol L, which exhibited the most significant activity among the remaining compounds. The chemical structure of SL is shown in Figure 1B. To assess its effect on GBM cells (LN-229 and T98G) and normal glial cells (SVGP12), cells were treated with increasing concentrations of SL for 48 hours. The results demonstrated that SL markedly inhibited cell growth even at relatively low concentrations. Moreover, the half-maximal inhibitory concentration (IC₅₀) of SL was significantly higher in SVGP12 cells than in LN-229 and T98G cells. Calculated IC₅₀ values were 16.51 μM in LN-229, 12.38 μM in T98G, and 73.06 μM in SVGP12 (Figure 1C). These findings demonstrated that SL, at its working concentration effective against tumor cells, did not harm normal glial cells. In further experiments, LN-229 and T98 G cells were treated with 10 μM or 20 μM SL for 48 h, respectively, using DMSO as the control. MTT assays confirmed that SL inhibited the proliferation of both GBM cell lines in a dose-dependent manner (Figure 1D). Consistent with this, microscopic examination showed a significant decrease in the number of viable LN-229 and T98 G cells with increasing SL concentrations (Figure 1E). *In vitro* colony-formation assays further revealed markedly reduced clonogenic ability in SL-treated cells compared with the control group (Figure 1F). Moreover, *in vivo* bioluminescence imaging of orthotopic xenografts demonstrated that tumors in SL-treated mice were significantly smaller than those in the control group (Figure 1G, Supplementary Figure S1B). SL treatment notably suppressed the tumorigenicity of GBM cells and prolonged the survival of tumor-bearing mice (Figure 1H). Importantly, as shown in Figure 1I, blood levels of transaminases and total protein showed no significant alterations before and after SL administration, indicating no apparent organ pathology or significant impairment of hepatic or renal function. Correspondingly, H&E staining was performed on the brain tissues of the orthotopic brain tumor-bearing mice. The results also demonstrated that the drug treatment had no discernible adverse effects on the normal brain tissue of the mice (Supplementary Figure S1C). The combined results indicate that SL displays low toxicity toward visceral organs in mice. These preliminary findings collectively demonstrate that SL effectively inhibits the proliferation of GBM cells both *in vitro* and *in vivo* without causing detectable liver or kidney injury, thereby highlighting its potential as a promising antitumor agent. Additionally, A comprehensive pharmacokinetic analysis was conducted. Following tail vein injection of SL in mice, blood samples were collected at predetermined time points for drug concentration measurement. Plasma concentrations were determined at 5 min, 15 min, 30 min, 1 h, 2 h, 4 h, 6 h, 12 h, and 24 h post-administration. The results revealed that the maximum plasma concentration (C_max_≈563 ng/mL) was achieved immediately after administration. The terminal elimination half-life (T1/2) was approximately 3.95 h (Supplementary Figure S1D). More pharmacokinetic data, such as drug release and tissue distribution, can be found in our previously study 13.

### SL can provoke cell cycle arrest and cytotoxic endoplasmic reticulum stress in GBM cells

**Figure 2:**
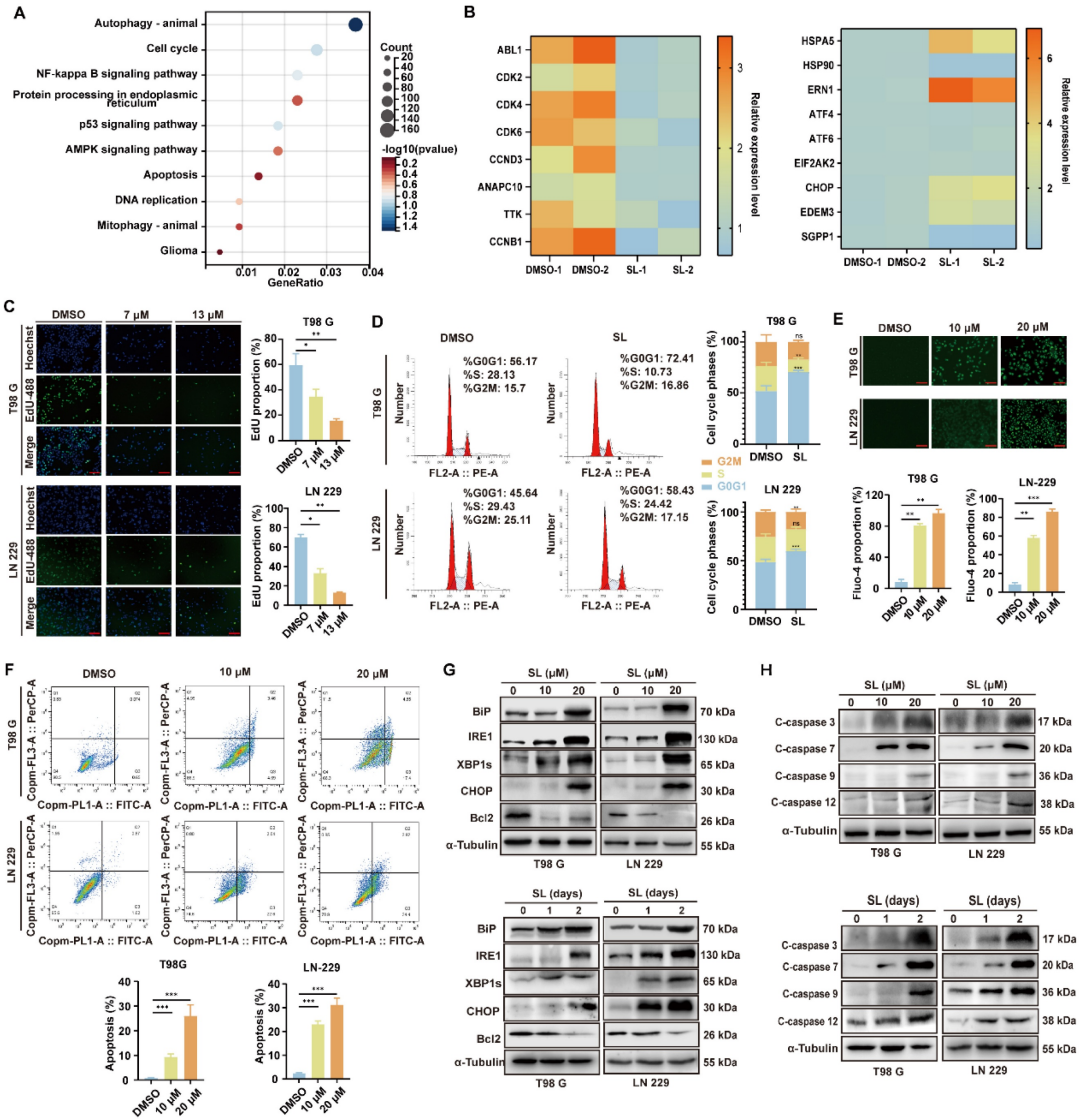
**Sanggenol L induced the cytotoxic ER stress in GBM cells and then leaded to apoptosis.** (**A**) KEGG analysis of downregulated or upregulated genes in LN-229 cells after SL treatment, as determined by proteomics data. DMSO-treated groups served as controls. (**B**) Heatmap showing the relative expression levels of selected ER-stress and cell-cycle-related genes based on proteomics data following SL treatment. The DMSO group was used as control. (**C**) EdU assay was performed to assess the proliferation capacity of GBM cells (T98G and LN-229) after 48-hour treatment with the indicated concentrations of SL. DMSO-treated groups served as controls. Scale bars = 200 μm. (**D**) Cell-cycle distribution in GBM cells (T98G and LN-229) was analyzed by flow cytometry after 48-hour treatment with the indicated concentrations of SL. DMSO-treated groups were used as controls. (**E**) Intracellular calcium levels in GBM cells (T98G and LN-229) treated with the indicated concentrations of SL for 48 hours were detected using Fluo-4 probe. DMSO-treated groups served as controls. Scale bars = 200 μm. (**F**) Apoptosis levels in GBM cells (T98G and LN-229) after 48-hour SL treatment at indicated concentrations were determined by flow cytometry. DMSO-treated groups were used as controls. (**G**) Western blot analysis of ER-stress-related proteins in GBM cells (T98G and LN-229). The upper panel shows results after treatment with a gradient concentration of SL for 48 hours; the lower panel shows results after treatment with 20 μM SL for different durations. DMSO-treated groups served as controls. (**H**) Western blot analysis of apoptosis-related proteins in GBM cells (T98G and LN-229). The upper panel shows results after treatment with a gradient concentration of SL for 48 hours; the lower panel shows results after treatment with 20 μM SL for different durations. DMSO-treated groups were used as controls. All data is shown as the means ± SD; n=3, *p < 0.05, **p < 0.01, ***p < 0.001.

To identify the key pathways or biological processes through which SL influences GBM cells, we conducted a proteomic analysis. Enrichment analysis of the quantitative proteomics data revealed significant enrichment of genes related to autophagy, cell cycle, and endoplasmic reticulum (ER) stress after SL treatment (Figure 2A, B; Supplementary Figure S2A, Supplementary Table S2). Among these, autophagy-related mechanisms have been established as a major research focus in other studies by our team 13. In contrast, this project primarily focused on the marked elevation of endoplasmic reticulum (ER) stress induced by SL (Figure 2B). We subsequently examined the effects of SL on cell cycle progression, ER stress, and apoptosis in GBM cells. EdU assay results showed that SL treatment significantly reduced cellular proliferation compared to the control (Figure 2C). As illustrated in Figure 2D, SL induced cell cycle arrest at the G0/G1 phase in both LN-229 and T98G cells. Further investigation revealed a dose-dependent increase in intracellular calcium levels upon SL treatment (Figure 2E). Moreover, flow cytometry analysis demonstrated that SL promoted apoptosis in a concentration-dependent manner in LN-229 and T98G cells (Figure 2F). To delineate the specific ER stress pathway activated by SL, we separately assessed three major signaling branches: IRE1, PERK, and ATF6. Western blot analysis indicated that only the IRE1 pathway was significantly upregulated. Consistent with this finding, SL treatment altered the expression of ER stress-related proteins in both a dose- and time-dependent manner (Figure 2G, Supplementary Figure S2B, C). Furthermore, the WB results showcased a clear dose-dependent and time-dependent upregulation of cleaved caspase proteins, including C-caspase 3, C-caspase 7, C-caspase 9, and C-caspase 12 (Figure 2H, Supplementary figure S2D, E). Moreover, mRNA quantification indicated that the increase occurred at the transcriptional level. This suggests a potential compensatory mechanism, in which elevated transcription of BiP and IRE1 is triggered as an adaptive attempt by the cells to mitigate cytotoxic ER stress (Supplementary figure S2F). These findings collectively indicate that SL suppresses glioblastoma cell proliferation through the induction of cell cycle arrest, while also promoting apoptosis via the activation of cytotoxic ER stress. These mechanisms underscoring the promising potential of SL as an anti-tumor agent.

### SL induced cytotoxic ER stress by specifically binding to BiP

**Figure 3:**
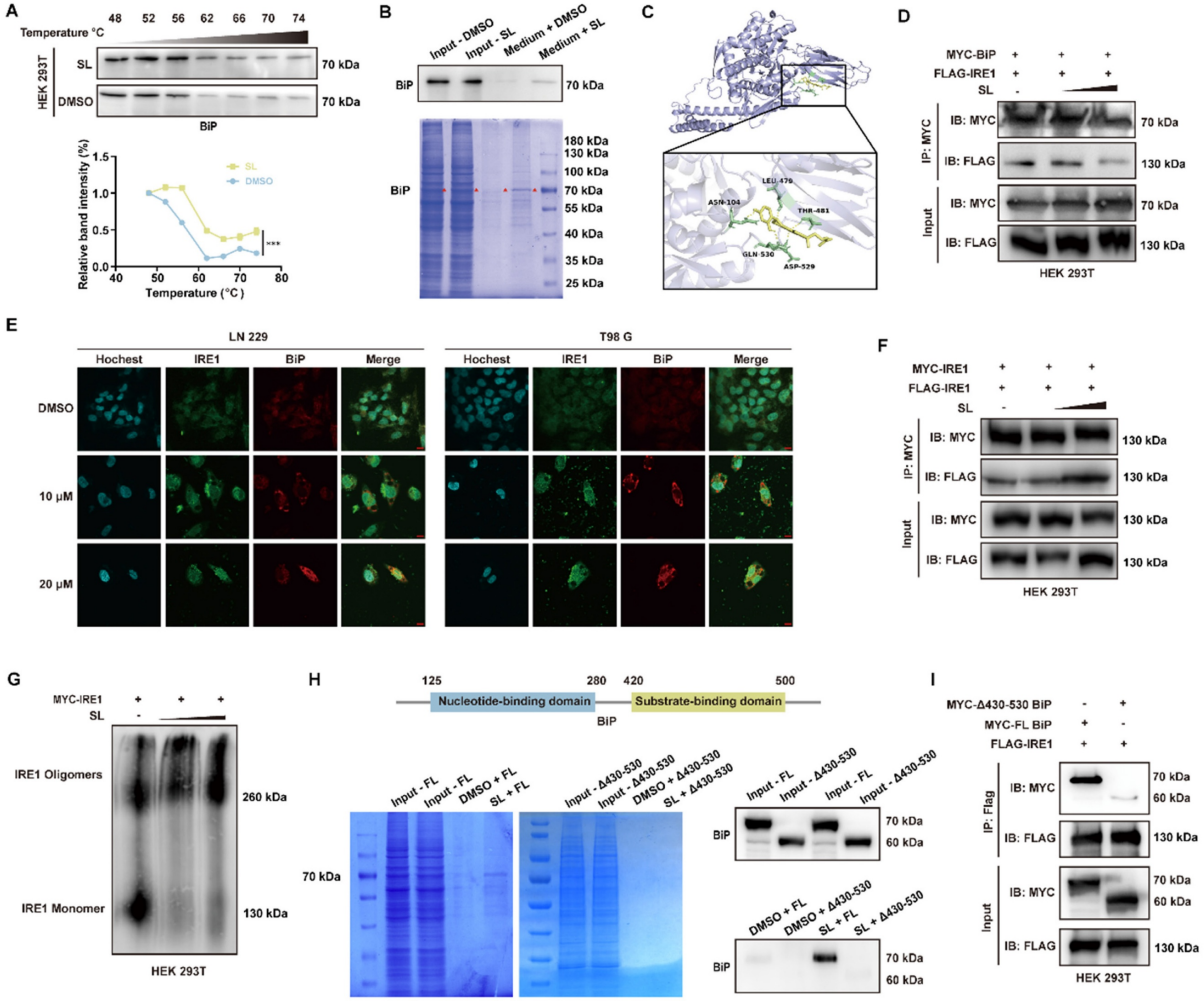
** BiP is the specific target protein of Sanggenol L.** (**A**) The CETSA assay was conducted in HEK 293T cells overexpressing BiP after treatment with a high concentration of SL for 3 hours. Western blot and quantitative analysis were used to determine whether the compound enhanced the thermal stability of the target protein, with the DMSO group serving as the control. (**B**) The interaction between SL and BiP was examined using a Sepharose 6B pull-down assay in HEK 293T cells overexpressing BiP. In the Coomassie brilliant blue staining figure, red triangles indicate the target protein BiP. The medium + DMSO group was used as the control. (**C**) The interaction between SL and BiP was predicted by AutoDock and visualized using PyMOL. The binding complex showed a binding energy of -8.26 kcal/mol. SL is shown in yellow, and the BiP protein structure is depicted in light blue. Specific BiP amino acids forming hydrogen bonds with SL (ASN-104, LEU-479, THR-481, ASP-529, and GLN-530) are highlighted in green. (**D**) HEK 293T cells overexpressing MYC-BiP and FLAG-IRE1 were treated with gradient concentrations of SL. MYC-BiP was pulled down using an anti-MYC antibody and immunoblotted with an anti-FLAG antibody, with the DMSO group as the control. (**E**) Following 48-hour treatment of GBM cells with SL or DMSO, immunofluorescence staining was performed to examine the localization of IRE1 and BiP, which was observed under a confocal microscope. DMSO groups served as controls. Scale bars = 10 μm. (**F**) HEK 293T cells overexpressing MYC-tagged and FLAG-tagged IRE1 were treated with gradient concentrations of SL. MYC-IRE1 was pulled down with an anti-MYC antibody and immunoblotted with an anti-FLAG antibody, using the DMSO group as the control. (**G**) Blue-Native PAGE was used to detect the level of IRE1 oligomers in HEK 293T cells overexpressing MYC-IRE1 after treatment with gradient concentrations of SL. DMSO groups were used as controls. (**H**) A schematic of the BiP protein domain structure is shown above. Sepharose 6B pull-down assays were performed in HEK 293T cells overexpressing full-length (FL) BiP or a mutant lacking amino acids 430-530 (Δ430-530). The FL group served as the control. (**I**) To identify the interaction domains between IRE1 and BiP, a BiP domain-deletion vector was used. The interaction of IRE1 with full-length (FL) or Δ430-530 BiP was assessed by co-immunoprecipitation, with the FL group as the control. All data is shown as the means ± SD; n=3, *p < 0.05, **p < 0.01, ***p < 0.001.

To elucidate the mechanism through which SL influences ER stress, we first sought to identify its direct protein target. An *in vitro* pull-down assay using epoxy-activated Sepharose 6B affinity media coupled with LC-MS/MS analysis revealed specific binding and enrichment of BiP (also known as HSPA5) by SL (Supplementary Table S3). This interaction was further validated by a cellular thermal shift assay (CETSA), which showed that SL increased the thermal stability of BiP (Figure 3A). Additionally, Western blot analysis following pull-down assays confirmed the specific binding between SL and BiP (Figure 3B). Together, these results indicate that the cytotoxic ER stress induced by SL in GBM cells is mediated through its direct targeting of BiP. We next investigated the functional link between SL binding and ER stress activation. Molecular docking simulations were performed to predict the potential binding site of SL on BiP. The results suggested a strong interaction between SL and the C-terminal region of the substrate-binding domain β (SBDβ) of BiP, with a calculated binding energy of -8.67 kcal/mol (Figure 3C, Supplementary Figure S3A). This region of BiP has been previously shown to be critical for its interaction with client proteins 15, 16. In addition, our previous results demonstrated that SL specifically upregulates the IRE1 signaling pathway. We therefore hypothesized that SL may bind to critical sites necessary for the IRE1-BiP interaction. Unbound IRE1 would then dimerize and initiate downstream signaling cascades 17, 18. To further investigate the binding of SL to BiP and its downstream consequences, BiP and IRE1 were overexpressed in HEK 293T cells. Western blot analysis revealed that increasing concentrations of SL promoted the dissociation of BiP from IRE1 (Figure 3D, Supplementary Figure S3B, C). Furthermore, immunofluorescence was employed to examine the effect of SL on the subcellular co-localization of BiP and IRE1. The results showed a clear alteration in protein localization even at low SL concentrations, indicating that SL disrupts the interaction between BiP and IRE1 (Figure 3E). This observation provided initial support for our hypothesis. Given that activation of the IRE1 signaling pathway is known to depend on its dimerization, we next assessed whether SL influences IRE1 dimer formation. Co-immunoprecipitation (Co-IP) experiments confirmed that the level of IRE1 dimer increased in a dose-dependent manner upon SL treatment (Figure 3F, Supplementary Figure S3C). Our hypothesis was further confirmed by consistent results from the Blue-Native PAGE analysis (Figure 3G, Supplementary Figure S3D, E).

Finally, to delineate the interaction domains between SL and BiP and between IRE1α and BiP, we constructed a deletion mutant. Pull-down assays and WB analysis showed that, in contrast to full-length (FL) BiP, SL failed to interact significantly with a BiP mutant lacking amino acids 430-530 (Figure 3H). Co-IP experiments similarly revealed that the binding between this mutant and IRE1α was substantially weakened. These results collectively indicate that the 430-530 amino acid region of BiP is essential for its interaction with both SL and IRE1α (Figure 3I). In summary, through pull-down, CETSA, and Co-IP assays, we demonstrate that SL specifically binds to the IRE1α-interaction site on BiP, thereby promoting BiP dissociation from IRE1α and increasing IRE1α dimerization. This action initiates cytotoxic ER stress dysregulation, which underlies the anti-glioblastoma effects of SL.

### SL induced cytotoxic ER stress in GBM by specifically targeting the BiP/IRE1 axis

**Figure 4:**
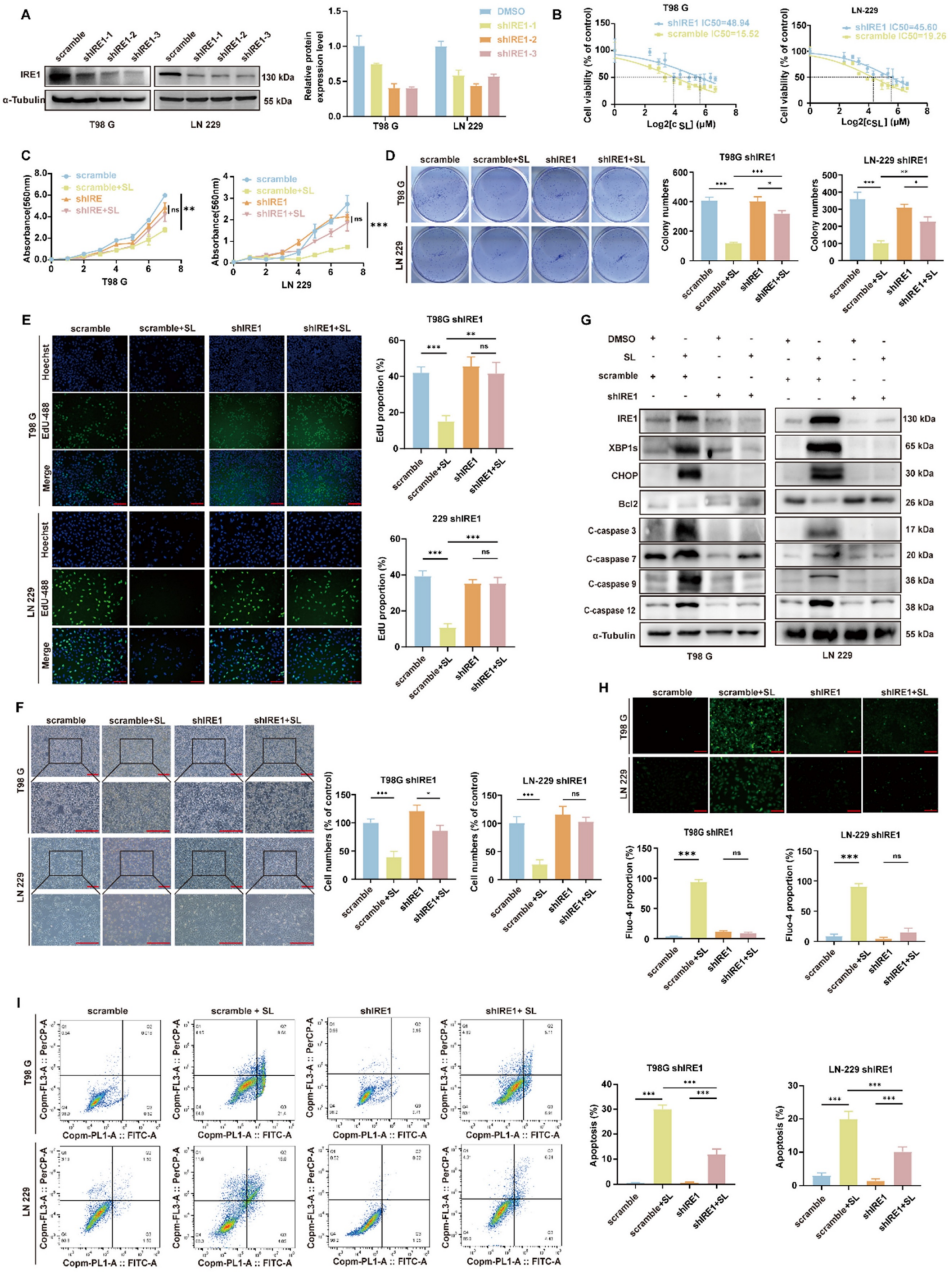
**The knockdown of IRE1 can partly reversed the function of Sanggenol L in GBM cells.** (**A**) Western blot analysis was performed to evaluate the interference efficiency of IRE1 in knockdown cell lines. The most effective line was selected to determine the IC₅₀ of SL. (**B**) The IC₅₀ of SL was 48.94 μM in T98G-shIRE1 and 45.60 μM in LN-229-shIRE1, whereas in normal glioma cell lines, the value was between 15 and 20 μM. Scramble groups served as controls. (**C**) The MTT assay was used to compare the inhibitory effect of SL or DMSO on cell proliferation in shIRE1 cell lines. Absorbance at 560 nm was measured after 1, 2, 3, 4, 5, 6, and 7 days of treatment. Scramble groups were used as controls. (**D**) Colony formation ability *in vitro* was assessed following treatment with SL or DMSO, and compared between normal GBM cells (T98G and LN-229) and shIRE1 cell lines. Scramble groups served as controls. (**E**) EdU assay was performed to evaluate the proliferation capacity of shIRE1 cell lines (T98G and LN-229) after 48 h of treatment with SL or DMSO. Scramble groups were used as controls. Scale bars = 200 μm. (**F**) Cell state and morphology of shIRE1 cell lines (T98G and LN-229) were observed under a microscope after treatment with SL or DMSO. Scramble groups served as controls. Scale bar = 200 μm. (**G**) Western blot was carried out to measure the levels of specific proteins in shIRE1 cell lines (T98G and LN-229) after 48 h of treatment with SL or DMSO. Scramble groups were used as controls. (**H**) Calcium ion levels in IRE1-knockdown GBM cells (T98G and LN-229) were detected using a Fluo-4 probe after 2 days of treatment with SL or DMSO. Scramble groups served as controls. Scale bar = 200 μm. (**I**) Apoptosis levels in shIRE1 cell lines (T98G and LN-229) were determined by flow cytometry after 48 h of treatment with SL or DMSO. Scramble groups were used as controls. All data is shown as the means ± SD; n=3, *p < 0.05, **p < 0.01, ***p < 0.001.

Based on the current findings, we hypothesized that IRE1α is a critical mediator of SL-induced cytotoxic ER stress and its consequent anti-proliferative effects. To test this, we generated stable IRE1α-knockdown cell lines via lentiviral infection (Figure 4A). Cell proliferation assays showed that IRE1α knockdown significantly increased the IC_50_ of SL in both LN-229 and T98G cells compared to controls (Figure 4B), underscoring the functional importance of IRE1α in SL's activity. Further evaluations, including MTT, colony formation, and EdU assays, confirmed that downregulation of IRE1α markedly enhanced the proliferation of SL-treated LN-229 and T98G cells (Figure 4C-E). It indicates that IRE1α knockdown partially rescued the cells from SL's anti-tumor effects. The microscopy also revealed that IRE1α silencing restored the normal morphology of SL-treated cells (Figure 4F). At the molecular level, western blot analysis demonstrated that IRE1α knockdown alleviated SL-induced alterations in protein levels related to ER stress and apoptosis (Figure 4G, Supplementary Figure S4A, B). Calcium flux assays indicated that IRE1α suppression significantly reduced the SL-triggered rise in intracellular Ca^2+^ (Figure 4H). Moreover, flow cytometry confirmed that IRE1α downregulation substantially decreased SL-induced apoptosis in both cell lines (Figure 4I). Collectively, these results establish IRE1α as a key effector through which SL exerts its anti-tumor functions, impacting GBM cell proliferation, ER stress, and apoptotic death. Specifically, the SL-BiP interaction appears to propagate its effects largely via the IRE1α signaling axis.

### SL triggered apoptosis in GBM cells through BiP/IRE1/CHOP/XBP1s/Bcl 2 axis

**Figure 5:**
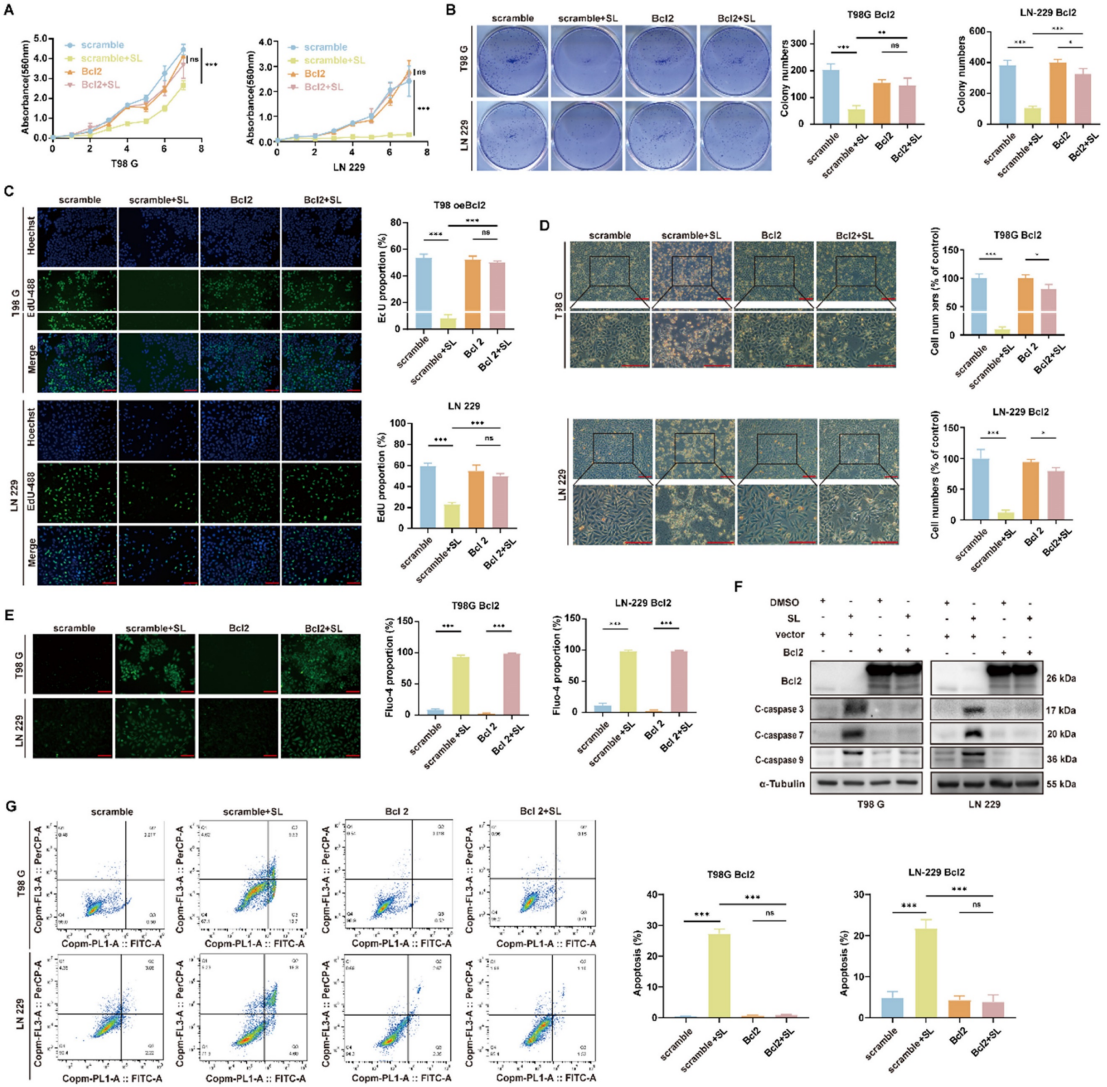
** The overexpression of Bcl2 can partly reversed the function of Sanggenol L in GBM cells.** (**A**) Cell viability in Bcl-2-overexpressing cell lines (T98G and LN-229) after treatment with SL or DMSO was assessed using the MTT assay. Absorbance at 560 nm was measured at 1, 2, 3, 4, 5, 6 and 7 days post-treatment. Scrambled groups served as controls. (**B**) The *in vitro* colony-forming ability of Bcl-2-overexpressing cell lines (T98G and LN-229) was evaluated following treatment with SL or DMSO. Scrambled groups were used as controls. (**C**) The proliferation capacity of Bcl-2-overexpressing cell lines (T98G and LN-229) after 48-hour treatment with SL or DMSO was determined by EdU assay. Scrambled groups served as controls. Scale bars = 200 μm. (**D**) Cell status and morphology of Bcl-2-overexpressing cell lines (T98G and LN-229) were examined under a microscope after treatment with SL or DMSO. Scrambled groups were used as controls. Scale bar = 200 μm. (**E**) Intracellular calcium ion levels in Bcl-2-overexpressing cell lines (T98G and LN-229) were detected using a Fluo-4 probe after 2 days of treatment with SL or DMSO. Scrambled groups served as controls. Scale bar = 200 μm. (**F**) Western blot analysis was performed to determine the expression levels of specific proteins in Bcl-2-overexpressing cell lines (T98G and LN-229) treated with SL or DMSO for 48 hours. Scrambled groups were used as controls. (**G**) Apoptosis levels in Bcl-2-overexpressing cell lines (T98G and LN-229) after 48-hour treatment with SL or DMSO were analyzed by flow cytometry. Scrambled groups served as controls. All data is shown as the means ± SD; n=3, *p < 0.05, **p < 0.01, ***p < 0.001.

The interaction between IRE1 and Bcl2 is widely recognized as a critical factor in the cellular response to ER stress 19, 20. Excessive activation of IRE1, which downregulates the anti-apoptotic protein Bcl2, constitutes a critical step in apoptosis induction. Therefore, to determine whether SL triggers apoptosis in GBM cells through IRE1 hyperactivation and subsequent Bcl2 suppression, we employed GBM cells overexpressing Bcl2. To clarify the role of Bcl2 in apoptosis downstream of SL-induced cytotoxic ER stress, normal GBM cells and overexpression cell lines were subjected to treatment with SL or DMSO. Findings from MTT, colony formation, and EdU assays consistently showed that Bcl2 overexpression significantly restored the proliferation of SL-treated LN-229 and T98G cells (Figure 5A-C).

Bright-field imaging further indicated that Bcl2 overexpression notably preserved the normal morphology of these cells (Figure 5D). In contrast, calcium flux assays revealed that Bcl2 overexpression did not affect the SL-induced increase in intracellular calcium levels (Figure 5E), suggesting that while Bcl2 counteracts SL-mediated suppression of proliferation, it does not significantly inhibit ER stress. Western blot analysis further confirmed that in SL-treated GBM cells, Bcl2 overexpression attenuated SL-induced alterations in the expression of downstream apoptosis-related proteins (Figure 5F; Supplementary Figure S5A, B). Moreover, flow cytometry analysis demonstrated that Bcl2 overexpression markedly reduced SL-triggered apoptosis in both LN-229 and T98G cells (Figure 5G). Collectively, these results indicate that Bcl2 functions as a downstream effector in SL-induced apoptosis and is not involved in modulating the accompanying ER stress. The findings support the conclusion that SL promotes apoptosis in GBM cells through over-activation of IRE1 and subsequent suppression of Bcl2.

In summary, our findings collectively demonstrate that BiP is the direct target of SL in GBM cells. By binding to the interaction interface between BiP and IRE1α, SL prevents their association and promotes the dimerization of unbound IRE1α. This accumulation of IRE1α dimers leads to hyperactivation of ER stress via the IRE1α/XBP1s/CHOP/Bcl2 signaling pathway, ultimately triggering apoptosis in GBM cells.

### SL enhanced the chemosensitivity of GBM cells to TMZ

**Figure 6:**
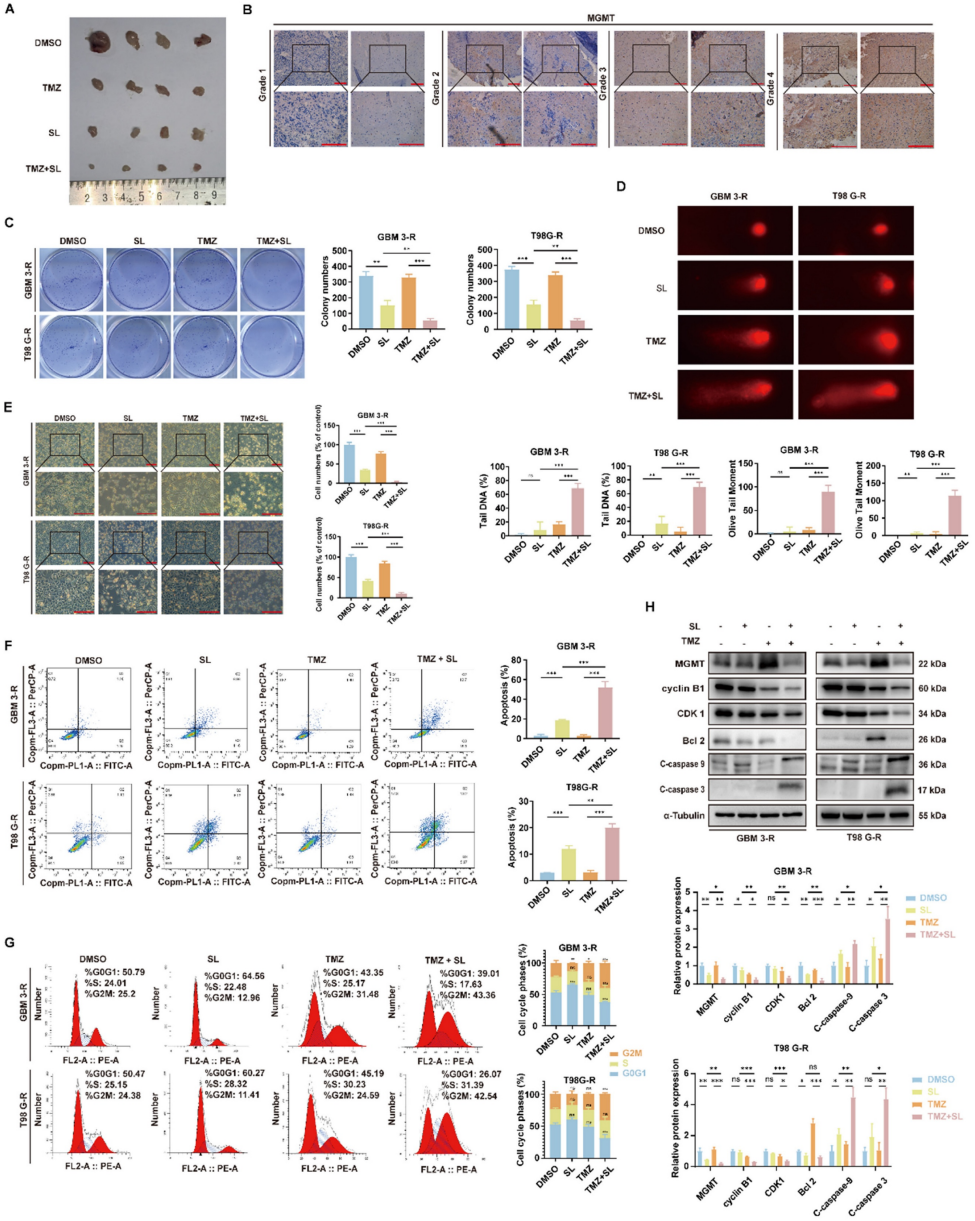
** Sanggenol L can promote the sensitivity of Temozolomide (TMZ) in GBM cells.** (**A**) One week after establishing subcutaneous GBM-3 tumors in mice, the animals were treated with SL, TMZ, or SL+TMZ, using DMSO as the control. When tumors in the DMSO group reached a large volume, the mice were euthanized and the tumors were excised for photographic documentation. n=4. (**B**) MGMT expression in tumor tissues from GBM patients was assessed by IHC staining. Glioma grade increases from left to right. Scale bar = 200 μm. (**C**) The effect of SL alone or in combination on colony formation was evaluated in TMZ-resistant cell lines (GBM-3-R and T98G-R), with DMSO groups serving as controls. (**D**) DNA damage in TMZ-resistant cell lines (GBM-3-R and T98G-R) following combination treatment was detected using the comet assay. DMSO groups were used as controls. (**E**) Cellular state and morphology of TMZ-resistant cell lines (GBM-3-R and T98G-R) were examined under a microscope after treatment with SL or combination therapy, with DMSO groups as controls. Scale bar = 200 μm. (**F**) Apoptosis levels in TMZ-resistant cell lines (GBM-3-R and T98G-R) treated with SL or combination therapy for 48 hours were determined by flow cytometry, using DMSO groups as controls. (**G**) Cell cycle distribution in TMZ-resistant cell lines (GBM-3-R and T98G-R) treated with SL or combination therapy for 48 hours was analyzed by flow cytometry, with DMSO groups as controls. (**H**) Expression levels of specific proteins in TMZ-resistant cell lines (GBM-3-R and T98G-R) treated with SL or combination therapy for 48 hours were determined by Western blot, using DMSO groups as controls. All data is shown as the means ± SD; n=3, *p < 0.05, **p < 0.01, ***p < 0.001.

Temozolomide (TMZ), an imidazotetrazine prodrug, is the first-line chemotherapeutic agent for glioblastoma (GBM). However, its clinical efficacy is frequently limited by poor patient response and the development of drug resistance 21. To explore strategies for overcoming TMZ resistance, we investigated whether and how SL could enhance the sensitivity of GBM cells to TMZ. First, using a subcutaneous xenograft model with the GBM-3 cell line in nude mice, we observed that the combination of TMZ and SL resulted in significantly smaller tumors compared to either monotherapy (Figure 6A), suggesting a potential synergistic therapeutic effect. The primary mechanism of TMZ resistance involves DNA repair, mediated notably by O^6^-methylguanine-DNA methyltransferase (MGMT). This enzyme directly repairs O^6^-methylguanine (O^6^-MeG) lesions induced by TMZ, thereby counteracting its cytotoxic effect. High MGMT expression is strongly associated with TMZ resistance 22. Consistently, immunohistochemical analysis of clinical tumor samples showed that MGMT expression increased with tumor grade (Figure 6B), correlating with heightened resistance. Subsequent *in vitro* experiments in TMZ-resistant GBM-3 and T98G cells demonstrated that the TMZ+SL combination markedly reduced colony formation (Figure 6C) and restored TMZ-induced DNA damage, as shown by comet assay (Figure 6D). Bright-field imaging further confirmed a more pronounced effect on cell growth and morphology with the combination treatment (Figure 6E). Flow cytometry analysis revealed that the combination significantly increased apoptosis (Figure 6F) and enhanced TMZ-induced cell cycle arrest (Figure 6G) compared to single agents. Western blot analysis corroborated these findings, showing that SL restored the ability of TMZ to modulate key cell cycle- and apoptosis-related proteins in resistant cells (Figure 6H). In summary, these results indicate that SL can enhance the chemosensitivity of TMZ-resistant glioblastoma cells to TMZ. The combination of SL and TMZ exerts a stronger inhibitory effect on GBM growth both *in vitro* and *in vivo*, highlighting its potential as a promising therapeutic strategy.

**Figure 7:**
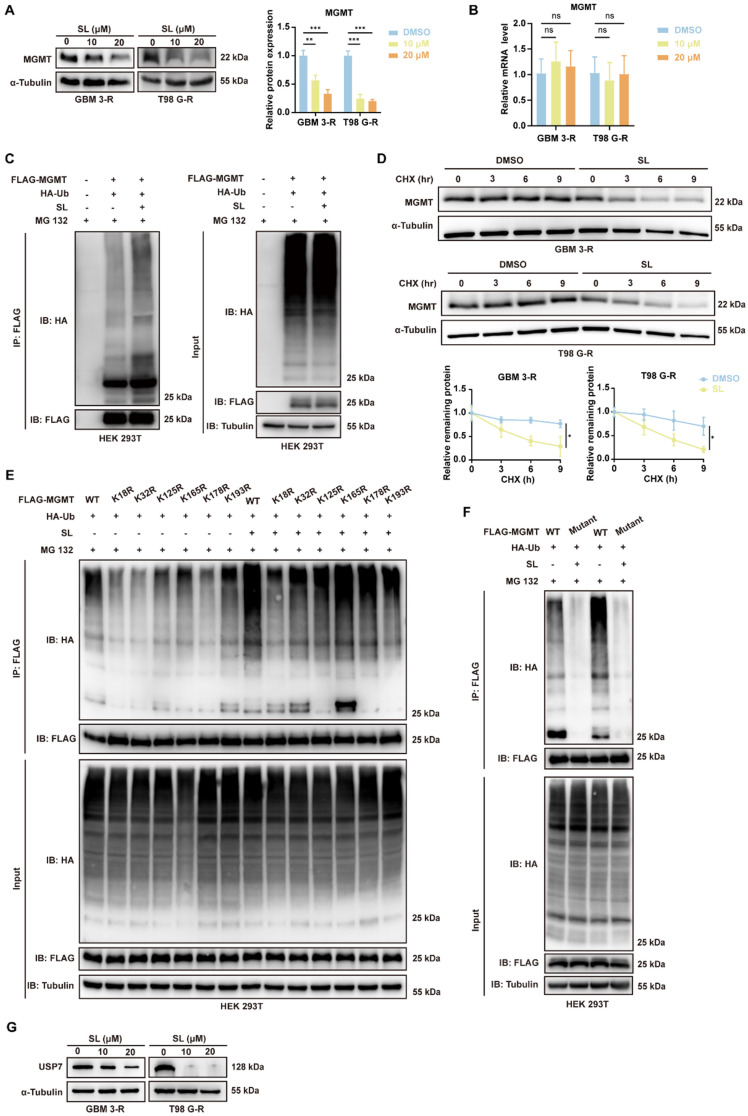
** Sanggenol L promoted the ubiquitination and degradation of MGMT by reducing USP7 levels.** (**A**) Western blot analysis was performed to assess MGMT protein levels in TMZ-resistant cell lines (T98G-R and GBM3-R) following treatment with the indicated concentrations of SL for 48 h. DMSO-treated groups served as controls. (**B**) The mRNA expression of MGMT in TMZ-resistant cell lines (T98G-R and GBM3-R) was evaluated by RT-qPCR after exposure to the specified concentrations of SL for 48 h. DMSO-treated groups were used as controls. (**C**) To examine the effect of SL on MGMT ubiquitination, HEK293T cells were transfected with the indicated plasmids and then treated with SL or DMSO. MG132 was added 8 h prior to cell lysis. Ubiquitination levels of MGMT were detected by Western blot. Cells transfected with empty vector and treated with DMSO served as controls. (**D**) TMZ-resistant cell lines (T98G-R and GBM3-R) were treated with SL (15 μM) or DMSO, followed by exposure to CHX (100 μg/mL) for the indicated durations. Cells were harvested and analyzed by Western blot to determine the turnover rate of MGMT. The 0-h time point was set as the baseline for each group, with DMSO treatment serving as the control. (**E**) MGMT expression vectors containing mutations at residues 18, 32, 125, 165, 178, or 193 were constructed. HEK293T cells were transfected with the respective plasmids and treated with SL or DMSO for 36 h. MG132 was added 8 h before cell collection. Key ubiquitination sites of MGMT were assessed by Co-IP. (**F**) MGMT expression vectors containing mutations at residues 18, 32, 125, 165, 178 and 193 were constructed. HEK293T cells were transfected with the respective plasmids and treated with SL or DMSO for 36 h. MG132 was added 8 h before cell collection. (**G**) USP7 protein expression in TMZ-resistant cell lines (T98G-R and GBM3-R) was detected by Western blot after treatment with the indicated concentrations of SL for 48 h. DMSO-treated groups were used as controls. All data is shown as the means ± SD; n=3, *p < 0.05, **p < 0.01, ***p < 0.001.

**Figure 8:**
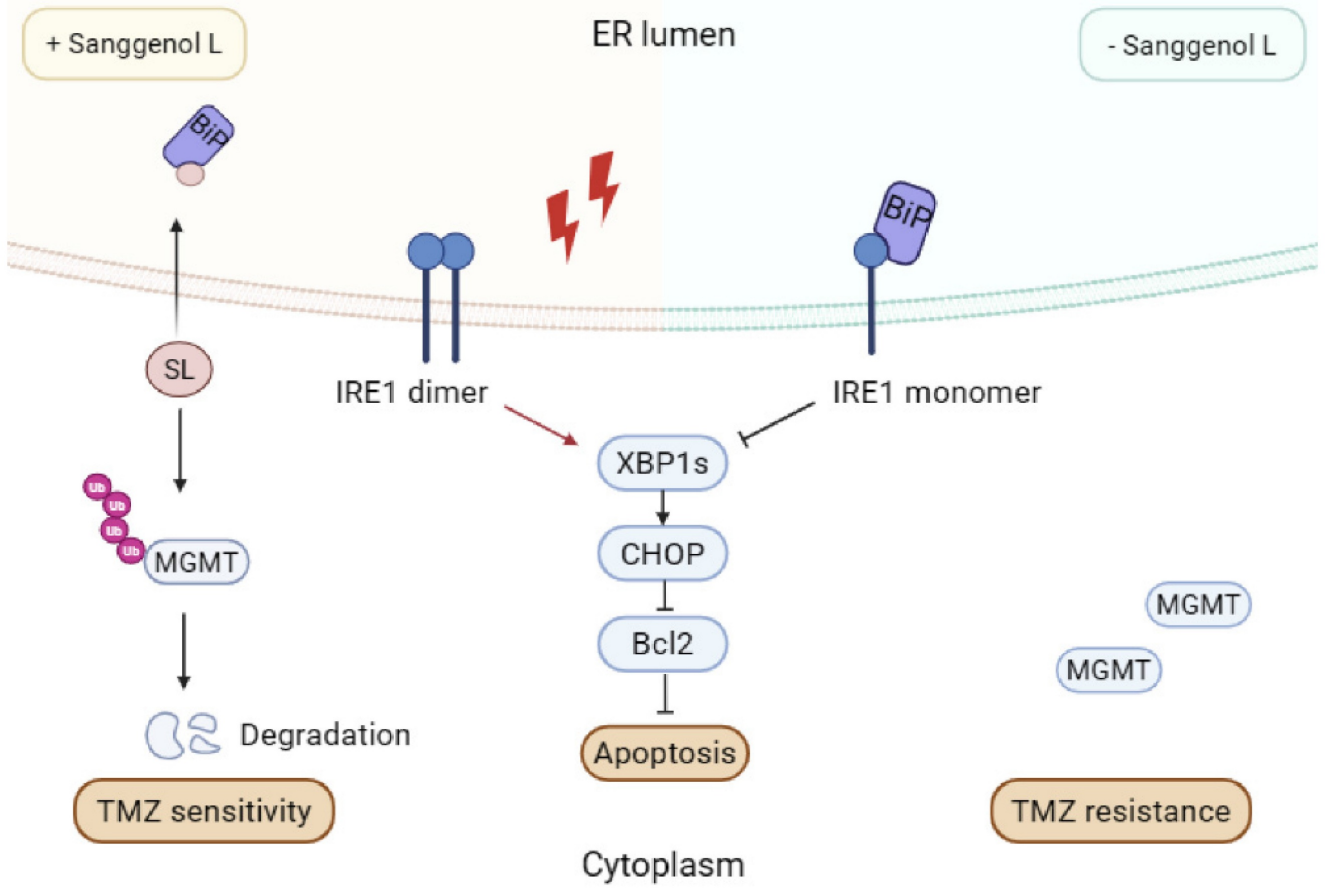
** The mechanism model diagram of Sanggenol L (SL).** SL binds to BiP and blocks its interaction with IRE1, consequently triggering cytotoxic endoplasmic reticulum stress through excessive activation of the IRE1/XBP1s/CHOP/Bcl2 signaling axis. Furthermore, SL potentiates TMZ sensitivity in GBM cells by facilitating MGMT ubiquitination and subsequent degradation.

Based on these findings, we explored the mechanism through which SL enhances the sensitivity of GBM cells to TMZ, focusing primarily on MGMT. Western blot analysis indicated that MGMT expression was markedly downregulated in TMZ-resistant GBM-3 and T98G cells treated with increasing concentrations of SL (Figure 7A). RT-qPCR results showed that SL treatment did not alter MGMT mRNA levels (Figure 7B), prompting us to investigate post-translational regulation. Ubiquitination and protein turnover assays confirmed that SL promotes MGMT ubiquitination and subsequent degradation, significantly reducing its half-life (Figure 7C, D, Supplementary figure S6A). To further clarify how Sanggenol L regulates MGMT ubiquitination, we investigated the types of ubiquitin chains involved and identified the specific ubiquitination sites on MGMT. The Co-IP and WB results showed that mutation at lysine 48 (K48) of ubiquitin led to a marked reduction in MGMT ubiquitination, whereas other ubiquitin mutants had no significant effect (Supplementary figure S6B). This indicates that MGMT ubiquitination primarily occurs via K48-linked polyubiquitin chains. Furthermore, based on publicly available mass spectrometry data from Cell Signaling Technology, potential ubiquitination sites on MGMT include amino acid residues 18, 32, 125, 165, 178, and 193 (Supplementary table S5) 23. Therefore, to investigate the key ubiquitination site(s) on MGMT through which SL exerts its effect, we constructed a series of MGMT expression vectors harboring mutations at residues 18, 32, 125, 165, 178, or 193. Co-IP assays performed after co-transfection of the mutant plasmids with HA-Ub into HEK 293T cells showed that each mutation influenced MGMT ubiquitination to varying degrees. Mutations at positions 18 and 32 had the most notable effects. Treatment with SL further highlighted the importance of lysine 18, as mutation at this site most strongly attenuated SL-induced ubiquitination of MGMT (Figure 7E). Together, these results suggest that SL predominantly enhances K48-linked ubiquitination at lysine 18 of MGMT. Additionally, we constructed an MGMT expression plasmid with mutations introduced at all six sites. As expected, this complete mutation abolished both the basal ubiquitination of MGMT and the capacity of SL to induce further ubiquitination (Figure 7F). Furthermore, we performed a preliminary exploration of the upstream proteins governing the MGMT ubiquitination process. Studies have indicated that USP7 and USP19 directly interact with MGMT and mediate its deubiquitylation 24, 25. Therefore, we examined the effect of SL on USP proteins by Western Blot analysis. The results revealed a significant downregulation of USP7 after SL treated (Figure 7G, Supplementary figure S6C). However, Sanggenol L showed no significant effect on USP19. We also confirmed the specific interaction between MGMT and USP7 (Supplementary figure S6D). Consistently, published studies indicate that USP7 regulates K48-linked ubiquitination, which aligns with our forementioned findings 26-28. In summary, these findings indicate that SL restores TMZ sensitivity in resistant cells by promoting K48-linked ubiquitination of MGMT at residue 18, mediated through the modulation of USP7.

## Discussion

As one of the gravest cancers globally, malignant brain tumors are characterized by a dismal prognosis. Patients further bear the additional and severe burden of significant deterioration in quality of life and cognitive faculties 29. Glioblastoma, which accounts for roughly 80% of malignant brain tumors, has an annual incidence of approximately 5.26 per 100,000 people, corresponding to about 17,000 new cases each year in the United States. It predominantly affects individuals aged 60 to 80 years, and its prevalence is anticipated to increase with the aging of the population 30-32. Despite decades of research, glioblastoma continues to portend a poor prognosis and significantly reduced quality of life. Although treatment methods have evolved, the median survival of 12-18 months represents only a slight extension beyond that achieved thirty years prior. A sobering testament to this limited progress is the five-year overall survival rate, which stands at approximately 5.1% 33. This lack of progress is primarily due to the absence of effective treatments alongside the formidable challenges inherent to drug development 34.

The standard GBM therapy of surgery, radiotherapy, and Temozolomide (TMZ) has yielded only modest extensions in survival, which fails to meet the urgent clinical need for more effective therapies 35. Temozolomide is an orally administered alkylating agent that crosses the blood-brain barrier and functions as both a pro-autophagic and pro-apoptotic agent. It is particularly effective against tumor cells exhibiting low O^6^-alkylguanine DNA alkyl transferase (OGAT) activity and deficient mismatch repair 36. While, MGMT overexpression is commonly implicated in the development of TMZ resistance, constituting a major barrier to effective treatment 37. Therefore, researchers are actively pursuing novel therapeutic strategies, which encompass targeted therapies, immunotherapies, and advanced drug delivery systems 38. For instance, molecular inhibitors targeting pathways such as EGFR, VEGF, and PI3K have exhibited promising efficacy in preclinical studies 39. Moreover, immunotherapies, including CAR-T cell therapy and immune checkpoint inhibitors, are being explored for their potential to stimulate an anti-tumor immune response, aiming to turn the body's own defenses against the cancer 40. Nevertheless, the efficacy of these advanced strategies is hindered by significant barriers, most notably the blood-brain barrier (BBB) and a profoundly immunosuppressive tumor microenvironment. The presence of the BBB often prevents delivered drugs from reaching effective concentrations within the central nervous system and at glioblastoma sites, posing a major obstacle to therapy. Consequently, current chemotherapeutic agents for glioma remain significantly limited. Accordingly, effectively extending patient survival requires the discovery of new therapeutic targets, a better elucidation of glioblastoma pathogenesis, and, critically, the development of novel treatments or drug delivery methods. In this context, the lipid nanoparticle (LNP) platform employed here constitutes a key enabling technology. LNPs can encapsulate therapeutic agents, facilitating their transit across the BBB and direct delivery to the CNS. Furthermore, their design can be tailored to target specific molecular features of tumors, enabling a personalized treatment approach 41.

The pivotal role of endoplasmic reticulum (ER) stress and the unfolded protein response (UPR) in oncology is increasingly recognized. ER stress, characterized by the accumulation of misfolded proteins within the ER, is commonly triggered by adverse conditions like hypoxia or nutrient deprivation. The ensuing UPR is an integrated adaptive response that seeks to mitigate stress and restore equilibrium. A key therapeutic aspect is that persistent, irremediable ER stress can pivot the UPR from a pro-survival to a pro-apoptotic outcome 42. BiP, a central ER chaperone, plays a vital role in UPR regulation by maintaining proteostasis and preventing its overactivation. It directly modulates the activity of IRE1, a key UPR sensor on the ER membrane. In the absence of severe stress, BiP binds to IRE1, inhibiting its oligomerization and autophosphorylation, which serves as a buffer against inappropriate UPR triggering. Conversely, when ER folding homeostasis is broken, BiP dissociates, allowing IRE1 to undergo oligomerization and activation. This ensures a timely resolution of the stress response. The dynamic nature of this interaction enables precise cellular adaptation to fluctuating ER stress 17.

Our study established that the compound SL specifically targets BiP. As demonstrated by LC-MS/MS, pull-down, and CETSA assays, this interaction creates a steric hindrance that disrupts the binding between BiP and IRE1α. In GBM cells, Sanggenol L consequently induces a state of chronically hyperactivated ER stress. As outlined earlier, the ER stress response plays a dual role: at basal or moderate levels, it serves as a protective, pro-survival mechanism. However, sustained pharmacological induction pushes this stress beyond the adaptive capacity of the cell, thereby switching it into a potent trigger for intrinsic apoptosis 43,44. SL induces apoptosis by specifically disrupting BiP function, thereby intensifying cytotoxic endoplasmic reticulum stress. Notably, this aligns with a prevailing strategy in anticancer drug development, whereby numerous agents (including various metal-based compounds and triphenylamine derivatives) are designed to intentionally hyperactivate ER stress pathways 45, 46. However, BiP itself has not been directly targeted in prior research. Our findings therefore highlight its potential as a therapeutic target and offer a promising new strategy for GBM treatment.

Beyond its potential as a monotherapy, SL also holds significant promise for combination strategies. Developing therapies that combine SL with existing treatments may represent the most effective approach to improving outcomes in GBM. A pivotal finding of our study is that SL enhances the chemosensitivity of GBM to TMZ by promoting K48-linked ubiquitination and subsequent degradation of MGMT. Furthermore, building upon prior evidence that USP7 interacts with and regulates the ubiquitination of MGMT, we have now identified the critical ubiquitination site at amino acid position 18. By demonstrating SL's role in this process, our work underscores the significance of the USP7-MGMT axis. Crucially, as MGMT overexpression is a primary driver of TMZ resistance, SL's ability to deplete MGMT levels presents a promising strategy to restore therapeutic efficacy. The dual mechanism of SL, modulating both ER stress and MGMT ubiquitination, highlights its potential as a compelling candidate for further development as an anticancer agent. Moving forward, greater consideration could be given to combination strategies. SL's dual mechanism supports its potential synergy with immunotherapy (checkpoint inhibitors) or targeted agents (anti-angiogenic drugs), especially since ER stress modulation can alter the tumor microenvironment and increase therapeutic susceptibility. This approach addresses the limited durability and adaptive resistance typical of GBM monotherapies. Presently, no standard therapeutics operate primarily via cytotoxic ER stress. Therefore, if future ER stress-inducing drugs are developed, co-administration with SL would represent a logical and compelling combination worthy of clinical evaluation.

Our findings suggest that SL represents a promising novel therapeutic candidate for GBM. However, several limitations of this study must be acknowledged. Mechanistically, while we demonstrated how SL induces cytotoxic endoplasmic reticulum stress and enhances chemosensitivity, the direct link between these two processes remains unclear. Specifically, further investigation is needed to determine whether SL's targeting of BiP and its effect on USP7 function operate sequentially or through additional regulatory components. On the other hand, translating Sanggenol L from a preclinical candidate to a clinically viable therapy requires addressing key translational challenges. Future work should focus on optimizing its administration and dosing, along with conducting thorough preclinical safety assessments and early-phase clinical trials to systematically evaluate efficacy and establish a favorable safety profile in GBM patients. Additionally, studies on its pharmacokinetic profile, including absorption, distribution, metabolism, and excretion (ADME) properties, are necessary to inform rational dosing regimens. Although we proposed a lipid nanoparticle-based delivery strategy that enhanced blood-brain barrier penetration and tumor targeting in a mouse model, further research is essential to advance this approach toward clinical translation. Finally, identifying predictive biomarkers of patient response to SL will be crucial for developing personalized treatment strategies.

In conclusion, our study elucidates key molecular mechanisms underlying GBM resistance and proposes a novel therapeutic strategy. By simultaneously targeting ER stress and chemotherapy sensitization, SL offers a dual-action approach to overcoming persistent challenges in GBM therapy. While further research and clinical validation are warranted, this work paves the way for developing more effective treatments.

## Supplementary Material

Supplementary figures and tables.

## Data Availability

The datasets used and/or analyzed during the current study are available from the corresponding author on reasonable request.

## Abbreviations

SL: Sanggenol L
GBM: glioblastoma
TMZ: Temozolomide
BiP: heavy-chain binding protein
HSPA5: heat shock protein family A member 5
IRE1: Inositol-requiring enzyme 1
XBP1s: spliced form of X-box binding protein 1
CHOP: C/EBP-homologous protein
BCL 2: B-cell lymphoma-2
MGMT: O-6-Methylguanine-DNA Methyltransferase
ERS: endoplasmic reticulu stress
DMSO: Dimethyl sulfoxide
MTT: Methylthiazolyldiphenyl-tetrazolium bromide
EdU: 5-Ethynyl-2'-deoxyuridine
H&E: Hematoxylin-Eosin
WB: Western Bolt
IHC: Immunohistochemistry
Co-IP: co-inmunoprecipitation
LC-MS/MS: liquid chromatography-tandem mass spectrometry
KEGG: Kyoto Encyclopedia of Genes and Genomes. SDS-PAGE, Sodium Dodecyl Sulfate-Polyacrylamide Gel Electrophoresis
BN-PAGE: Blue/Clear Native-Polyacrylamide Gel Electrophoresis
CETSA: Cell Thermal Shift Assay
