# Supplementary material for: Sanggenol L Induces Endoplasmic Reticulum Stress and Triggers Cell Apoptosis in Glioblastoma by Binding to BiP
Source: Int J Biol Sci. 2026 Apr 16;22(9):4517–36. doi: 10.7150/ijbs.126892 (PMC13181853; doi:10.7150/ijbs.126892)
Supplement: Supplementary file 1 — Supplementary figures and tables. [file ijbsv22p4517s1.pdf]

Supplementary Materials for

Sanggenol L Induces Endoplasmic Reticulum Stress and Triggers Cell Apoptosis in Glioblastoma by Binding to BiP

Jingyang Xu *et al.*

*Corresponding author. Email: hcui@swu.edu.cn; hongjuan.cui@gmail.com

Supplementary Table S1. Table of Correspondence between the NO. and Name of the Mulberry Active Substances:

Table of Correspondence between the NO. and Name of the Mulberry Active Substances	
NO.	Molecule Name
S1-1	DMSO
S1-2	Dimethyl (methylenedi-4,1-phenylene)biscarbamate
S1-3	moracenin D
S1-4	limonianin
S1-5	ursolic acid
S1-6	oxyresveratrol-2-O-β-D-glucopyranoside
S2-1	Morusin
S2-2	sanggenol L
S2-3	kuwanon A
S2-4	Kuwanon S
S2-5	Moracenin
S2-6	3'-geranyl-3-prenyl-2',4',5,7-tetrahydroxyflavone
S3-1	sanggenone C
S3-2	moracin M-3'-O-β-D-glucopyranoside
S3-3	Oxysanguinarine
S3-4	moracin O
S3-5	morusignin L
S3-6	sanggenol A
S4-1	Mulberrin
S4-2	sanggenone D
S4-3	sanggenol N
S4-4	mulberroside C
S4-5	moracenin C
S4-6	kuwanon Y
S5-1	Juglanin
S5-2	albanol B
S5-3	7-methoxy-5,4'-dihydroxyflavanonol
S5-4	76464-71-6
S5-5	1'-Methoxy-2'-hydroxydihydromollugin
S5-6	Sitogluside
S6-1	Iristectorigenin (9CI)
S6-2	mulberranol
S6-3	Mairin
S6-4	sanggenone B
S6-5	sanguinarine
S6-6	oleanolic acid
S7-1	sanggenone N
S7-2	mulberrofuran M

S7-3	mulberrofuran G
S7-4	tectorigenin
S7-5	sanggenone M
S7-6	kuwanon H
S8-1	sanggenone H
S8-2	Moracin C
S8-3	Moracin D
S8-4	kuwanon G
S8-5	Morusinol
S8-6	morin
S9-1	cyclomulberrin
S9-2	kuwanon B
S9-3	(+)-Cycloolivil
S9-4	quercetin
S9-5	kuwanon D
S9-6	Kaempferol
S10-1	myrianthic acid
S10-2	Mulberrofuran C
S10-3	Sexangularetin
S10-4	Avicularin
S10-5	Glabrone
S10-6	myricetin

Supplementary Table S2. The most regulated gene in proteomic (Top 10):

	Upregulated		Downregulated	
	Gene name	Fold change (SL/DMSO)	Gene name	Fold change (SL/DMSO)
LN-229	PTGS2	312.0069188	MSMO1	0.003240663
	AP4E1	189.4740235	AFAP1	0.003951582
	MRPL41	186.1956786	PLP1	0.00750475
	PCOLCE	140.3822647	ATAD2	0.008035239
	IFT57	113.848266	BDH2	0.009433773
	APOE	96.6256964	DGKZ	0.012085283
	DBNDD1	78.84782339	ST3GAL4	0.012759097
	TGFBI	60.69959976	SDF4	0.014034529
	NDUFB4	59.8158156	APCDD1L	0.015440987
	GSTCD	51.38679181	ERBB3	0.015790814
T98 G	PLEKHA2	407.5887801	CLIC5	0.001119036
	CSF3	241.0735797	JAM2	0.003660995
	TULP3	181.9160651	ZDHHC20	0.006844668
	EEF1AKMT2	111.0736182	FDFT1	0.008372808
	TUT1	98.48274542	CYSTM1	0.012569698
	QSOX1	85.8814597	ZDHHC13	0.015141708
	PRUNE1	82.69359104	MRPS21	0.017711185
	RAB43	73.13782711	MIEN1	0.017845981
	GTPBP2	71.51958523	DIAPH3	0.020078609
	CFB	70.43332451	ZNF706	0.021363254

Supplementary Table S3. The most significant proteins in LC-MS/MS (Top 10):

Gene name	Sequence coverage-SL [%]	iBAQ (SL/DMSO)
HSPA5	51.4	4.58464934
SBSN	48.6	1.228005998
ALDOC	44.2	1.096500797
DPYSL2	43.1	0.984617491
KPRP	42.5	7.272099448
SNAP25	42.3	1.052086825
YWHAQ	38.3	1.105750351
GNAO1	36.7	0.359955883
IGKC	31.2	1.256259003
HBB	30.2	0.560575181

Supplementary Table S4. The shRNA sequences were listed as below:

shIRE1α-1-F	CCGGCCCATCAACCTCTCTTCTGTACTCGAGTACAGAAGAGAGGTTGATGGGTTTTTG
shIRE1α-1-R	AATTCAAAAACCCATCAACCTCTCTTCTGTACTCGAGTACAGAAGAGAGGTTGATGGG
shIRE1α-2-F	CCGGGAAATACTCTACCAGCCTCTACTCGAGTAGAGGCTGGTAGAGTATTTCTTTTTG
shIRE1α-2-R	AATTCAAAAAGAAATACTCTACCAGCCTCTACTCGAGTAGAGGCTGGTAGAGTATTTC
shIRE1α-3-F	CCGGCCTGCTTAATGTCAGTCTACACTCGAGTGTAGACTGACATTAAGCAGGTTTTTG
shIRE1α-3-R	AATTCAAAAACCTGCTTAATGTCAGTCTACACTCGAGTGTAGACTGACATTAAGCAGG

Supplementary Table S5. The potential ubiquitination sites on MGMT:

GENE	PROTEIN	ORGANISM	ACC#	GENE_ID	MW_(DA)	SITE_GRP_ID	RSD	MOD_TYPE	SITE_+/-7_AA
MGMT	MGMT	human	P16455	4255	21,646	983947847	M1	N-Ubi	_______mDKDCEMk
MGMT	MGMT	human	P16455	4255	21,646	964527000	K18	Ubiquityl	tLDsPLGkLELsGCE
MGMT	MGMT	human	P16455	4255	21,646	964527004	K32	Ubiquityl	EQGLHEIkLLGKGTS
MGMT	MGMT	human	P16455	4255	21,646	12365630	K125	Ubiquityl	AALAGNPkAARAVGG
MGMT	MGMT	human	P16455	4255	21,646	573761820	K165	Ubiquityl	ySGGLAVkEWLLAHE
MGMT	MGMT	human	P16455	4255	21,646	15392697	K178	Ubiquityl	HEGHRLGkPGLGGSS
MGMT	MGMT	human	P16455	4255	21,646	964527002	K193	Ubiquityl	GLAGAWLkGAGATSG

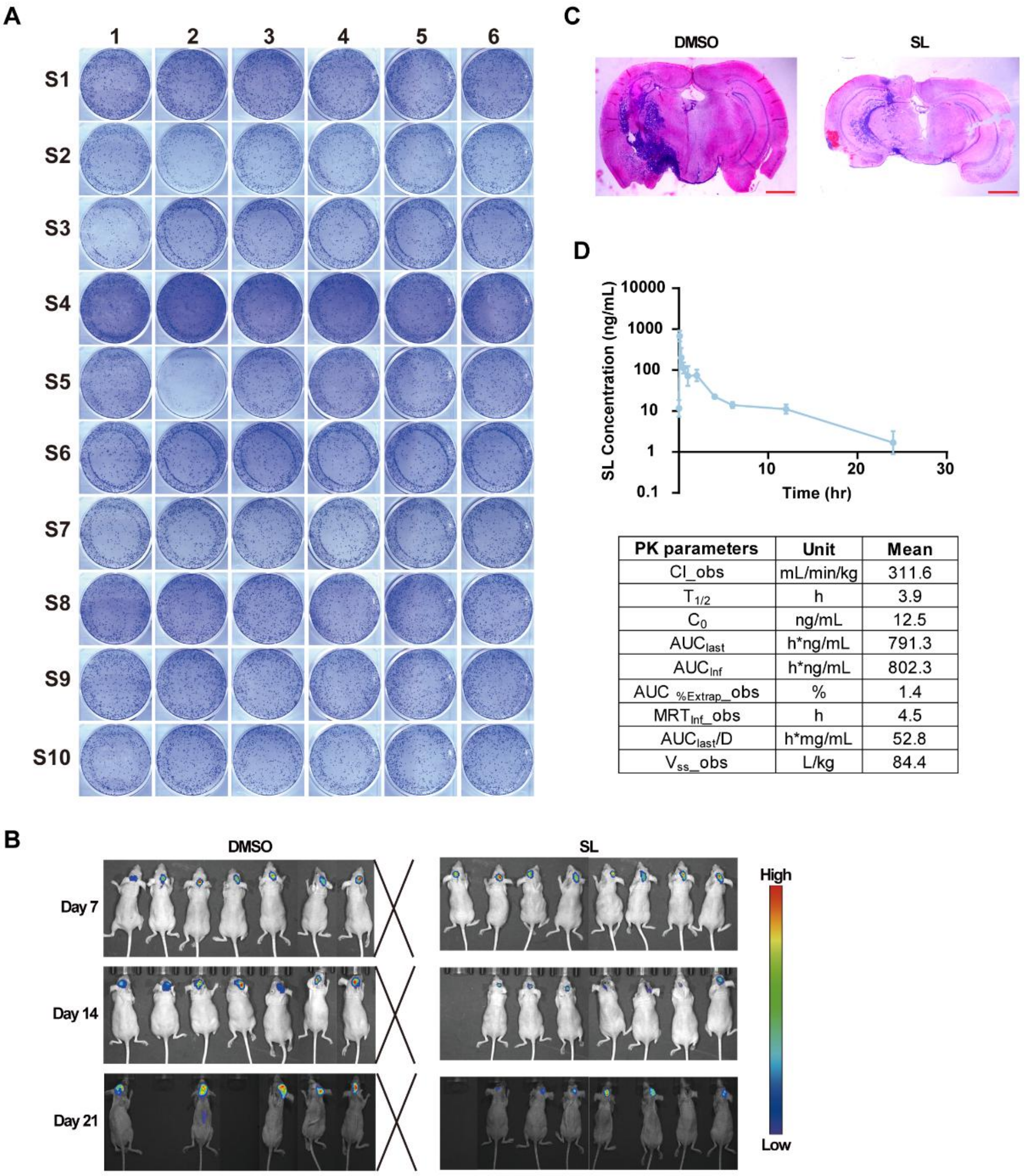

PK parameters	Unit	Mean
Cl_obs	mL/min/kg	311.6
$T_{1/2}$	h	3.9
C_0	ng/mL	12.5
AUC_{last}	h*ng/mL	791.3
AUC_{Inf}	h*ng/mL	802.3
$AUC_{\%Extrap_}$obs	%	1.4
$MRT_{Inf_}$obs	h	4.5
AUC_{last}/D	h*mg/mL	52.8
$V_{ss_}$obs	L/kg	84.4

Supplementary Figure S1. (A) Through the plate cloning experiment, 59 active substances from mulberry were used to treat GBM cells (LN-229) at a concentration of 15 μM. DMSO was used as control. (B) *In vivo* imaging was performed to evaluate the therapeutic effects of the SL (30 mg/kg) in mice with orthotopic transplanted tumor. The soybean phosphatidylcholine, cholesterol, DSPE-PEG2k-Biotin, DSPE-PEG2k-HA, and IR-780 were used to encapsulate SL and it was administrated by intravenous injection. n=8. (C) H&E staining of brain tissue from mice with orthotopically implanted tumors. DMSO was used as control. Scale bars= 2 mm. n=8 (D) Following tail vein injection of Sanggenol L, drug concentrations in mice blood samples were analyzed at 5 min, 15 min, 30 min, 1 h, 2 h, 4 h, 6 h, 12 h, and 24 h post-administration. Subsequent pharmacokinetic analysis was performed. n=3.

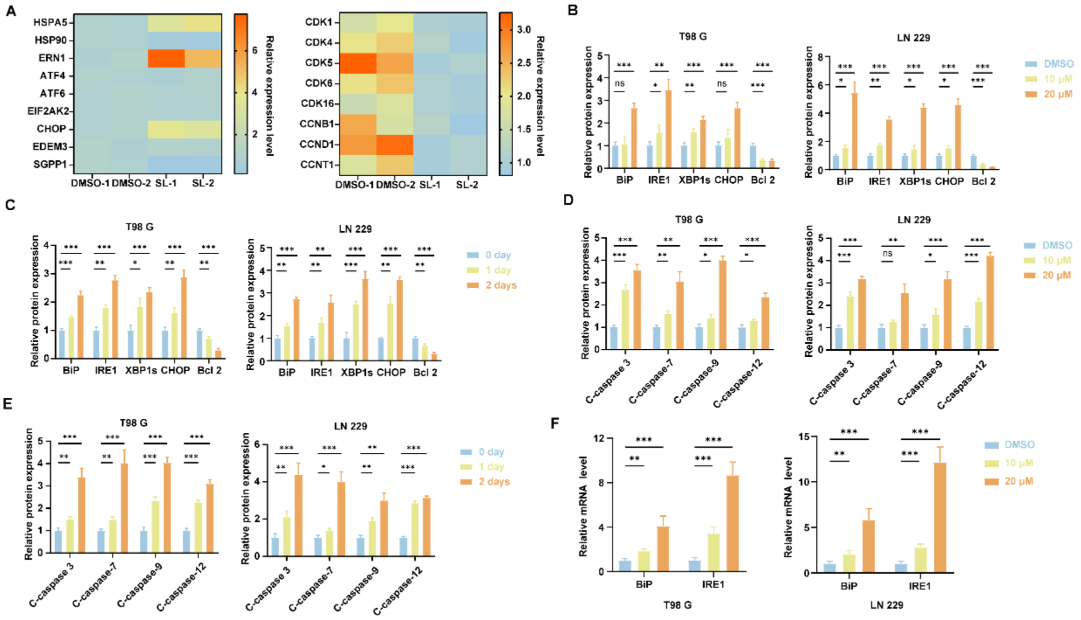

Supplementary Figure S2. (A) According to the proteomics data, heat map was used to visualize the relative expression level of some ER stress and cell cycle related gene after treated with SL. The DMSO group was used as control. (B) Present the quantitative results of figure 2G (above) in the form of a bar chart. (C) Present the quantitative results of figure 2G (below) in the form of a bar chart. (D) Present the quantitative results of figure 2H (above) in the form of a bar chart. (E) Present the quantitative results of figure 2H (below) in the form of a bar chart. (F) RT-qPCR assays were performed to evaluate the mRNA expression level of BiP and IRE1 in GBM cells. The DMSO groups were used as control.

All data are shown as the means ± SD; n=3, *p < 0.05, **p < 0.01, ***p < 0.001.

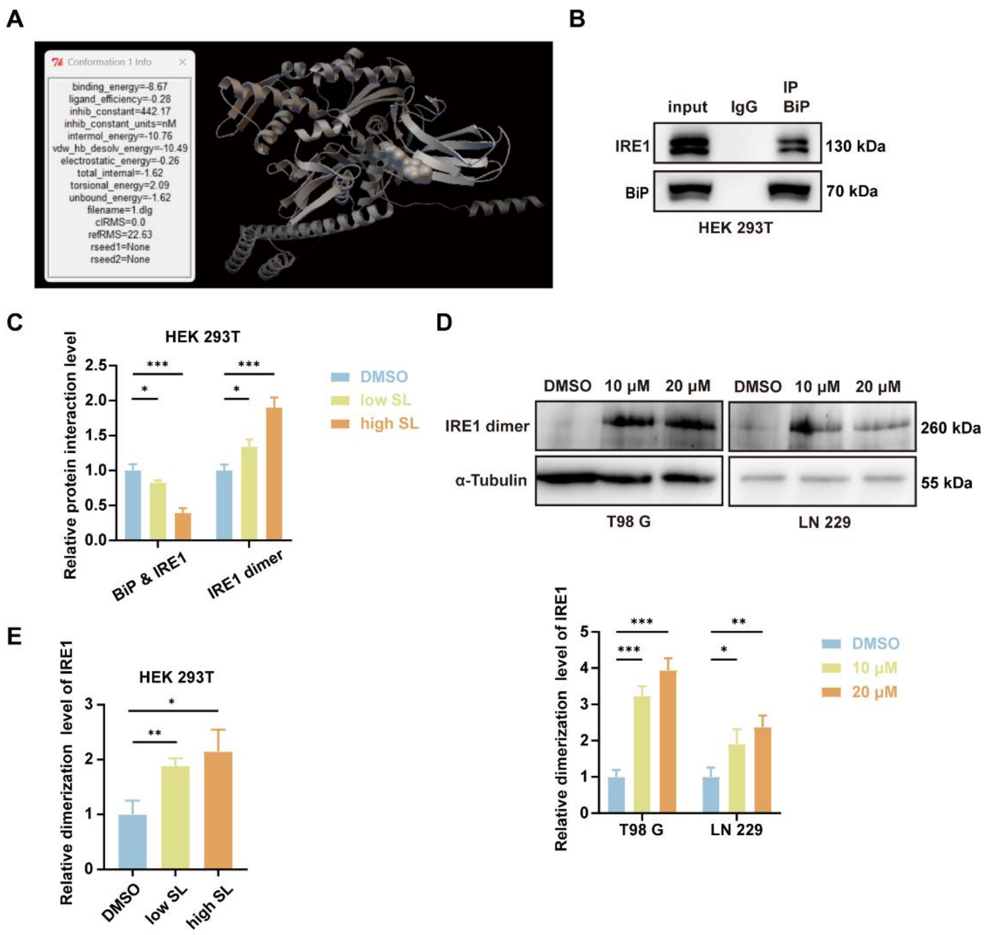

Supplementary Figure S3. (A) The molecular docking analysis of SL with BiP using AutoDock yielded a binding energy of -8.67 kcal/mol. (B) A specific interaction between IRE1 and BiP was observed. (C) Present the quantitative results of figure 3D, F in the form of a bar chart. (D) The Blue-Native PAGE Running assay was used to detect the level of IRE1 dimer in GBM cells (T98 G and LN-229) after treated with indicated concentration of SL. The expression levels of α-Tubulin were detected through SDS-PAGE Running assay. The DMSO groups were used as control. (E) Present the quantitative results of figure 3G in the form of a bar chart.

All data are shown as the means ± SD; n=3, *$p < 0.05$, **$p < 0.01$, ***$p < 0.001$.

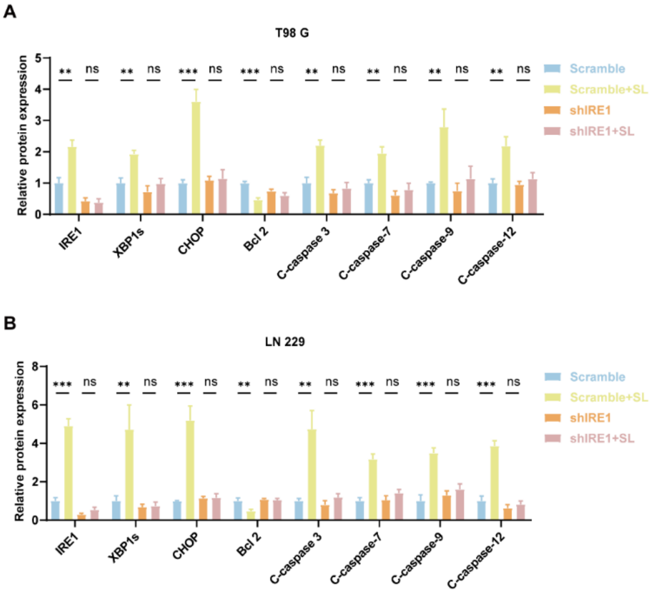

Supplementary Figure S4. (A) Present the quantitative results of figure 4G (T98 G) in the form of a bar chart. (B) Present the quantitative results of figure 4G (LN 229) in the form of a bar chart. All data are shown as the means ± SD; n=3, *p < 0.05, **p < 0.01, ***p < 0.001.

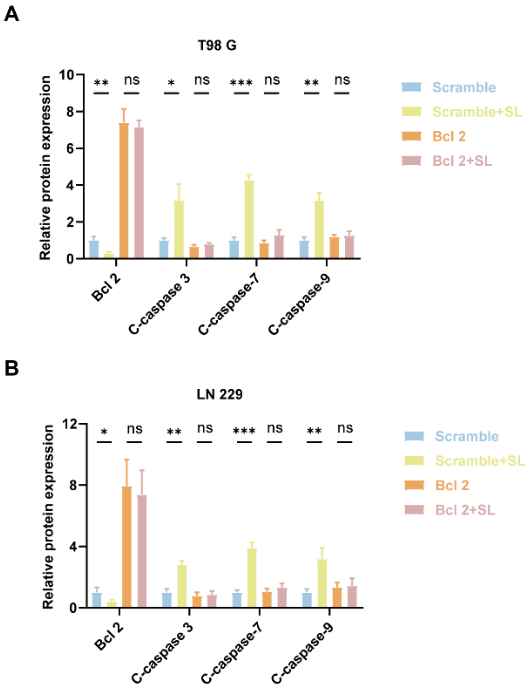

Supplementary Figure S5. (A) Present the quantitative results of figure 5F (T98 G) in the form of a bar chart. (B) Present the quantitative results of figure 5F (LN 229) in the form of a bar chart. All data are shown as the means ± SD; n=3, *p < 0.05, **p < 0.01, ***p < 0.001.

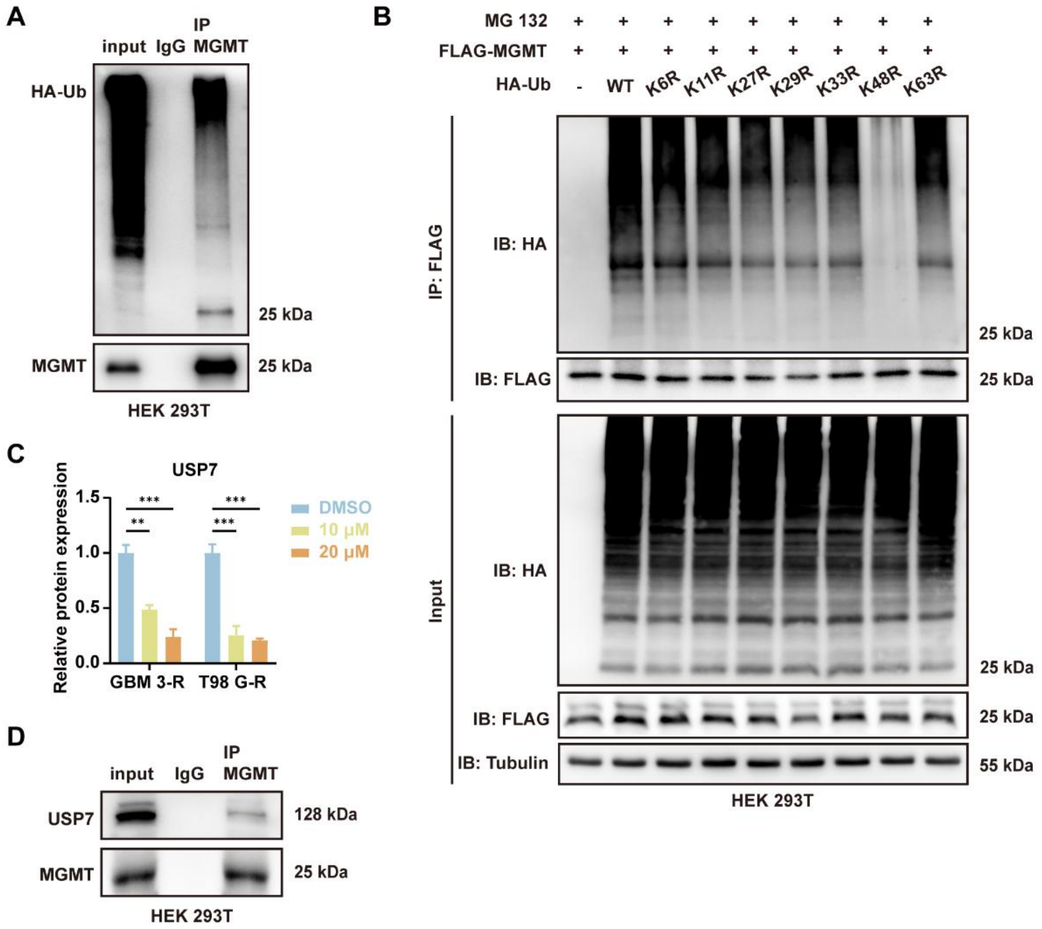

Supplementary Figure S6. (A) A specific interaction between the ubiquitin chain and MGMT was observed. (B) Ubiquitin expression vectors with mutations at designated sites were constructed. The specified plasmids were transfected into HEK 293T cells respectively, and the ubiquitin chain linkage on MGMT was subsequently detected by Co-IP. MG132 was added 8 hours before cell harvesting. (C) Present the quantitative results of figure 7G in the form of a bar chart. (D) A specific interaction between MGMT and USP7 was observed.

All data are shown as the means ± SD; n=3, $*p < 0.05$, $**p < 0.01$, $***p < 0.001$.

Details of the statistical methods and statistical analysis results

Figure 1 To detect the effect of Sanggenol L on the proliferation of glioblastoma.

(**D**) T98 G, DMSO vs 10 μM, p<0.0001, F=47.01, DMSO vs 20 μM, p<0.0001, F=43.32; LN-229, DMSO vs 10 μM, p<0.0001, F=39.27, DMSO vs 20 μM, p<0.0001, F=40.91, Two-way ANOVA (or mixed model).
(**E**) T98 G, DMSO vs 10 μM: p=0.0061, t=12.76, DMSO vs 20 μM: p=0.001, t=32.12; LN-229, DMSO vs 10 μM: p=0.0361, t=5.12, DMSO vs 20 μM: p=0.0086, t=10.73; t-test.
(**F**) T98 G, DMSO vs 10 μM: p=0.0163, t=7.729, DMSO vs 20 μM: p=0.0083, t=10.93; LN-229, DMSO vs 10 μM: p=0.0163, t=7.743, DMSO vs 20 μM: p=0.0047, t=14.57; t-test.
(**H**) DMSO vs SL: p=0.0004, Chi square=12.78; Cox proportional-hazards model.
(**I**) ALB, DMSO group: p=0.2849, t=1.13, SL group: p=0.5693, t=0.5885; GPT/ALT, DMSO group: p=0.4913, t=0.7145, SL group: p=0.4965, t=0.7056; GOT/AST, DMSO group: p=0.2124, t=1.332, SL group: p=0.1841, t=1.427; TP, DMSO group: p=0.9628, t=0.0479, SL group: p=0.9485, t=0.0663; t-test.

Figure 2 Sanggenol L induced the cytotoxic ER stress in GBM cells and then leaded to apoptosis.

(**C**) T98 G, DMSO vs 7 μM: p=0.0253, t=4.156, DMSO vs 13 μM: p=0.0028, t=6.574; LN-229, DMSO vs 7 μM: p=0.0104, t=9.734, DMSO vs 13 μM: p=0.0014, t=26.28; t-test.
(**D**) T98 G, DMSO vs SL, F=26.88, GOG1 phase: p=0.0002, S phase p=0.0057, G2M phase: p=0.2199; LN-229, DMSO vs SL, F=21.49, GOG1 phase: p=0.0007, S phase p=0.0735, G2M phase: p=0.0096; Two-way ANOVA (or mixed model).
(**E**) T98 G, DMSO vs 10 μM: p=0.0016, t=24.84, DMSO vs 20 μM: p=0.0021, t=21.58; LN-229, DMSO vs 10 μM: p=0.0022, t=21.34, DMSO vs 20 μM: p=0.001, t=31.95; t-test.
(**F**) T98 G, DMSO vs 10 μM: p=0.0003, t=11.98, DMSO vs 20 μM: p=0.0007, t=9.596; LN-229, DMSO vs 10 μM: p=0.001, t=24.43, DMSO vs 20 μM: p=0.001, t=17.44; t-test.

Figure 3 BiP is the specific target protein of Sanggenol L

(**A**) DMSO vs SL, p<0.0001, F=42.88; Two-way ANOVA (or mixed model).

Figure 4 The knockdown of IRE1 can partly reversed the function of Sanggenol L in GBM cells

(**C**) T98 G, scramble vs scramble+SL, p<0.0001, F=69.84, shIRE1 vs shIRE1+SL, p=0.2604, F=1.349; LN-229, scramble vs scramble+SL, p<0.0001, F=49.56, shIRE1 vs shIRE1+SL, p=0.0635, F=6.725, Two-way ANOVA (or mixed model).
(**D**) T98 G, scramble vs scramble+SL, p<0.0001, t=20, shIRE1 vs shIRE1+SL, p=0.0158, t=4.025, scramble+SL vs shIRE1+SL, p<0.0001, t=17.7; LN-229, scramble vs scramble+SL, p=0.0005, t=10.41, shIRE1 vs shIRE1+SL, p=0.0142, t=4.153, scramble+SL vs shIRE1+SL, p=0.0023, t=6.929, t-test.
(**E**) T98 G, scramble vs scramble+SL, p=0.0005, t=10.28, shIRE1 vs shIRE1+SL, p=0.438, t=0.8607, scramble+SL vs shIRE1+SL, p=0.0027, t=6.592; LN-229, scramble vs scramble+SL, p=0.0002, t=13.77, shIRE1 vs shIRE1+SL, p=0.9, t=0.01, scramble+SL vs shIRE1+SL, p=0.0005, t=10.33, t-test.
(**F**) T98 G, scramble vs scramble+SL, p=0.001, t=8.65, shIRE1 vs shIRE1+SL, p=0.0129, t=4.273; LN-229, scramble vs scramble+SL, p=0.001, t=8.627, shIRE1 vs shIRE1+SL, p=0.2319, t=1.408,

t-test.
(**H**) T98 G, scramble vs scramble+SL, $p<0.0001$, t=38.41, shIRE1 vs shIRE1+SL, p=0.1286, t=1.911; LN-229, scramble vs scramble+SL, $p<0.0001$, t=24.05, shIRE1 vs shIRE1+SL, p=0.0535, t=2.711, t-test.
(**I**) T98 G, scramble vs scramble+SL, $p<0.0001$, t=29.42, shIRE1 vs shIRE1+SL, p=0.0008, t=9.018, scramble+SL vs shIRE1+SL, p=0.0003, t=11.41; LN-229, scramble vs scramble+SL, p=0.0003, t=11.71, shIRE1 vs shIRE1+SL, p=0.0006, t=9.793, scramble+SL vs shIRE1+SL, p=0.0036, t=6.118, t-test.

Figure 5 The overexpression of Bcl2 can partly reversed the function of Sanggenol L in GBM cells

(**A**) T98 G, scramble vs scramble+SL, $p<0.0001$, F=40.18, Bcl2 vs Bcl2+SL, p=0.2604, F=1.28; LN-229, scramble vs scramble+SL, $p<0.0001$, F=34.05, Bcl2 vs Bcl2+SL, p=0.4768, F=0.9592, Two-way ANOVA (or mixed model).
(**B**) T98 G, scramble vs scramble+SL, p=0.0005, t=10.35, Bcl2 vs Bcl2+SL, p=0.6067, t=0.5579, scramble+SL vs Bcl2+SL, p=0.0075, t=5.007; LN-229, scramble vs scramble+SL, p=0.0002, t=13.29, Bcl2 vs Bcl2+SL, p=0.0352, t=3.129, scramble+SL vs Bcl2+SL, p=0.0006, t=10.01, t-test.
(**C**) T98 G, scramble vs scramble+SL, $p<0.0001$, t=21.49, Bcl2 vs Bcl2+SL, p=0.2466, t=1.356, scramble+SL vs Bcl2+SL, $p<0.0001$, t=25.5; LN-229, scramble vs scramble+SL, $p<0.0001$, t=20.29, Bcl2 vs Bcl2+SL, p=0.2427, t=1.37, scramble+SL vs Bcl2+SL, $p<0.0001$, t=17.05, t-test.
(**D**) T98 G, scramble vs scramble+SL, $p<0.0001$, t=17.43, Bcl2 vs Bcl2+SL, p=0.0267, t=3.425; LN-229, scramble vs scramble+SL, p=0.0005, t=10.41, Bcl2 vs Bcl2+SL, p=0.025, t=3.494, t-test.
(**E**) T98 G, scramble vs scramble+SL, $p<0.0001$, t=42.12, Bcl2 vs Bcl2+SL, $p<0.0001$, t=121.5; LN-229, scramble vs scramble+SL, $p<0.0001$, t=39.87, Bcl2 vs Bcl2+SL, $p<0.0001$, t=135, t-test.
(**G**) T98 G, scramble vs scramble+SL, $p<0.0001$, t=30.86, Bcl2 vs Bcl2+SL, p=0.0684, t=2.478, scramble+SL vs Bcl2+SL, $p<0.0001$, t=30.38; LN-229, scramble vs scramble+SL, p=0.0004, t=11.03, Bcl2 vs Bcl2+SL, p=0.7187, t=0.3866, scramble+SL vs Bcl2+SL, p=0.0004, t=11.19, t-test.

Figure 6 Sanggenol L can promote the sensitivity of Temozolomide (TMZ) in GBM cell

(**C**) GBM 3-R, DMSO vs SL: p=0.0015, t=7.74, TMZ vs TMZ+SL: $p<0.0001$, t=21.29, SL vs TMZ+SL: p=0.0087, t=4.792; T98 G-R, DMSO vs SL: p=0.0003, t=11.62, TMZ vs TMZ+SL: $p<0.0001$, t=21.99, SL vs TMZ+SL: p=0.0031, t=6.393, t-test.
(**D**) Tail DNA (%), GBM 3-R, DMSO vs SL: p=0.2029, t=1.363, TMZ vs TMZ+SL: $p<0.0001$, t=15.79, SL vs TMZ+SL: $p<0.0001$, t=10.61; T98 G-R, DMSO vs SL: p=0.0027, t=3.963, TMZ vs TMZ+SL: $p<0.0001$, t=16.96, SL vs TMZ+SL: $p<0.0001$, t=10.54; Olive Tail Moment, GBM 3-R, DMSO vs SL: p=0.2478, t=1.227, TMZ vs TMZ+SL: $p<0.0001$, t=13.55, SL vs TMZ+SL: $p<0.0001$, t=12.31; T98 G-R, DMSO vs SL: p=0.002, t=5.849, TMZ vs TMZ+SL: $p<0.0001$, t=15.63, SL vs TMZ+SL: $p<0.0001$, t=16.22, t-test.
(**E**) GBM 3-R, DMSO vs SL: $p<0.0001$, t=17.29, TMZ vs TMZ+SL: $p<0.0001$, t=20.68, SL vs TMZ+SL: $p<0.0001$, t=18.79; T98 G-R, DMSO vs SL: p=0.0001, t=14.88, TMZ vs TMZ+SL: $p<0.0001$, t=21.31, SL vs TMZ+SL: p=0.0003, t=11.67, t-test.
(**F**) GBM 3-R, DMSO vs SL: p=0.0002, t=12.67, TMZ vs TMZ+SL: p=0.0002, t=13.7, SL vs TMZ+SL: p=0.0007, t=9.392; T98 G-R, DMSO vs SL: p=0.0002, t=12.67, TMZ vs TMZ+SL: $p<0.0001$, t=19.26, SL vs TMZ+SL: p=0.0016, t=7.584, t-test.

(**G**) GBM 3-R, F=43.09, DMSO vs SL, GOG1 phase: p=0.0003, S phase p=0.6066, G2M phase: p=0.007, DMSO vs TMZ, GOG1 phase: p=0.2805, S phase p=0.5641, G2M phase: p=0.0215, DMSO vs TMZ+SL, GOG1 phase: p<0.0001, S phase p=0.8781, G2M phase: p<0.0001; T98 G-R, F=27.43, DMSO vs SL, GOG1 phase: p=0.0893, S phase p=0.9953, G2M phase: p=0.1403, DMSO vs TMZ, GOG1 phase: p=0.6673, S phase p=0.8956, G2M phase: p=0.9704, DMSO vs TMZ+SL, GOG1 phase: p<0.0001, S phase p=0.4926, G2M phase: p<0.0001, Two-way ANOVA (or mixed model).
(**H**) GBM 3-R, DMSO vs SL, MGMT: p=0.0054, t=5.471, cyclin B1: p=0.0416, t=2.958, CDK 1: p=0.1159, t=2.002, Bcl2: p=0.0077, t=4.958, C-caspase 9: p=0.0109, t=4.495, C-caspase 3: p=0.024, t=3.54; TMZ vs TMZ+SL, MGMT: p=0.0017, t=7.477, cyclin B1: p=0.0118, t=4.393, CDK 1: p=0.016, t=4.009, Bcl2: p=0.0008, t=9.106, C-caspase 9: p=0.002, t=7.155, C-caspase 3: p=0.0056, t=5.429; SL vs TMZ+SL, MGMT: p=0.0138, t=4.19, cyclin B1: p=0.0029, t=6.524, CDK 1: p=0.0025, t=6.726, Bcl2: p=0.0062, t=5.276, C-caspase 9: p=0.0234, t=3.569, C-caspase 3: p=0.0311, t=3.259; T98 G-R, DMSO vs SL, MGMT: p=0.0075, t=4.992, cyclin B1: p=0.4199, t=0.898, CDK 1: p=0.074, t=2.404, Bcl2: p=0.0277, t=3.382, C-caspase 9: p=0.019, t=3.802, C-caspase 3: p=0.1543, t=1.754; TMZ vs TMZ+SL, MGMT: p=0.0004, t=10.69, cyclin B1: p<0.0001, t=25.73, CDK 1: p=0.0241, t=3.535, Bcl2: p=0.0002, t=12.82, C-caspase 9: p=0.0013, t=8.076, C-caspase 3: p=0.003, t=6.435; SL vs TMZ+SL, MGMT: p=0.0015, t=7.695, cyclin B1: p=0.0004, t=10.75, CDK 1: p=0.0002, t=14.93, Bcl2: p=0.2283, t=1.421, C-caspase 9: p=0.0046, t=5.733, C-caspase 3: p=0.0202, t=3.735, t-test.

Figure 7 Sanggenol L promoted the ubiquitination and degradation of MGMT by reducing USP7 levels

(**A**) GBM 3-R, DMSO vs 10 μM: p=0.0044, t= 5.794, DMSO vs 20 μM: p=0.0006, t=9.889; T98 G-R, DMSO vs 10 μM: p=0.0003, t=12, DMSO vs 20 μM: p<0.0001, t=15.75, t-test.
(**B**) GBM 3-R, DMSO vs 10 μM: p=0.4376, t= 0.8614, DMSO vs 20 μM: p=0.6084, t=0.5551; T98 G-R, DMSO vs 10 μM: p=0.6216, t=0.534, DMSO vs 20 μM: p=0.9387, t=0.0819, t-test.
(**D**) GBM 3-R, DMSO vs SL: p=0.0218, F=4.248; T98 G-R, DMSO vs SL: p=0.0371, F=3.592, Two-way ANOVA (or mixed model).

Supplementary Figure S2.

(**B**) T98 G, DMSO vs 10 μM, BiP: p=0.765, t=0.3204, IRE1: p=0.0428, t=2.557, XBP1s: p=0.0041, t=5.913, CHOP: p=0.189, t=1.577, Bcl2: p=0.0009, t=8.765; DMSO vs 20 μM: BiP: p=0.0004, t=11.27, IRE1: p=0.0011, t=8.528, XBP1s: p=0.0005, t=10.49, CHOP: p=0.0006, t=9.954, Bcl2: p=0.0009, t=8.784; LN-229, DMSO vs 10 μM, BiP: p=0.0161, t=3.995, IRE1: p=0.0017, t=7.451, XBP1s: p=0.0461, t=2.856, CHOP: p=0.0138, t=4.183, Bcl2: p=0.0005, t=10.6; DMSO vs 20 μM: BiP: p=0.0006, t=9.774, IRE1: p<0.0001, t=19.39, XBP1s: p<0.0001, t=22.23, CHOP: p=0.0002, t=14.13, Bcl2: p<0.0001, t=16.14; t-test.
(**C**) T98 G, 0 d vs 1 d, BiP: p=0.0003, t=11.57, IRE1: p=0.0011, t=8.347, XBP1s: p=0.0182, t=3.854, CHOP: p=0.0081, t=4.894, Bcl2: p=0.0046, t=5.744; 0 d vs 2 d: BiP: p=0.0002, t=14.63, IRE1: p=0.0002, t=15.00, XBP1s: p=0.0006, t=9.984, CHOP: p=0.0003, t=11.88, Bcl2: p=0.0002, t=15.46; LN-229, 0 d vs 1 d, BiP: p=0.0046, t=5.698, IRE1: p=0.0065, t=5.186, XBP1s: p=0.0007, t=9.404, CHOP: p=0.0011, t=8.538, Bcl2: p=0.0057, t=5.393; 0 d vs 2 d, BiP: p=0.0006, t=19.91, IRE1: p=0.0014, t=7.924, XBP1s: p<0.001, t=10.98, CHOP: p<0.0001, t=33.25, Bcl2: p=0.0002, t=12.51;

t-test.

(**D**) T98 G, DMSO vs 10 μM, C-Caspase 3: p=0.0003, t=11.65, C-Caspase 7: p=0.0038, t=6.041, C-Caspase 9: p=0.0336, t=3.178, C-Caspase 12: p=0.017, t=3.934; DMSO vs 20 μM: C-Caspase 3: p<0.0001, t=15.76, C-Caspase 7: p=0.0014, t=7.892, C-Caspase 9: p<0.0001, t=22.92, C-Caspase 12: p=0.0003, t=11.74; LN-229, DMSO vs 10 μM, C-Caspase 3: p=0.0004, t=11.29, C-Caspase 7: p=0.0515, t=2.747, C-Caspase 9: p=0.0317, t=3.238, C-Caspase 12: p=0.0002, t=12.75; DMSO vs 20 μM: C-Caspase 3: p<0.0001, t=27.74, C-Caspase 7: p=0.0031, t=6.356, C-Caspase 9: p=0.0005, t=10.43, C-Caspase 12: p<0.0001, t=35.79; t-test.

(**E**) T98 G, 0 d vs 1 d, C-Caspase 3: p=0.0061, t=5.294, C-Caspase 7: p=0.0049, t=5.61, C-Caspase 9: p=0.0008, t=8.994, C-Caspase 12: p<0.0001, t=16.07; 0 d vs 2 d, C-Caspase 3: p=0.0006, t=9.613, C-Caspase 7: p=0.0009, t=8.773, C-Caspase 9: p<0.0001, t=17.2, C-Caspase 12: p<0.0001, t=17.99; LN-229, 0 d vs 1 d, C-Caspase 3: p=0.0088, t=4.77, C-Caspase 7: p=0.0257, t=3.461, C-Caspase 9: p=0.0022, t=7.003, C-Caspase 12: p<0.0001, t=20.63; 0 d vs 2 d, C-Caspase 3: p=0.0008, t=9.102, C-Caspase 7: p=0.0007, t=9.255, C-Caspase 9: p=0.0011, t=8.38, C-Caspase 12: p<0.0001, t=31.95; t-test.

(**F**) T98 G, DMSO vs 10 μM, BiP: p=0.0043, t=6.776, IRE1: p=0.0002, t=7.643; DMSO vs 20 μM, BiP: p=0.0005, t=6.978, IRE1: p<0.0001, t=12.77; LN-229, DMSO vs 10 μM, BiP: p=0.0046, t=4.404, IRE1: p=0.0003, t=7.171; DMSO vs 20 μM, BiP: p=0.0002, t=7.782, IRE1: p<0.0001, t=13.19; t-test.

Supplementary Figure S3.

(**C**) DMSO vs low SL, BiP & IRE1: p=0.0354, t=3.122, IRE1 dimer: p=0.0107, t=4.571; DMSO vs high SL, BiP & IRE1: p=0.0008, t=9.273, IRE1 dimer: p=0.0007, t=9.335; t-test.

(**D**) T98 G, DMSO vs 10 μM, p=0.0002, t=11.85, DMSO vs 20 μM, p=0.0002, t=13.4; LN-229, DMSO vs 10 μM, p=0.0322, t=3.221, DMSO vs 20 μM, p=0.0043, t=5.833; t-test.

(**E**) DMSO vs low SL, p=0.0061, t=5.302, DMSO vs high SL, p=0.0138, t=4.189; t-test.

Supplementary Figure S4.

(**A**) Scramble vs Scramble+SL, IRE1: p=0.0017, t=7.462, XBP1s: p=0.0014, t=7.835, CHOP: p=0.0004, t=11.25, Bcl2: p=0.0004, t=11.26, C-caspase 3: p=0.0011, t=8.448, C-caspase 7: p=0.0022, t=6.987, C-caspase 9: p=0.0056, t=5.423, C-caspase 12: p=0.0034, t=6.211; shIRE1 vs shIRE1+SL, IRE1: p=0.6371, t=0.4543, XBP1s: p=0.161, t=1.717, CHOP: p=0.8094, t=0.2576, Bcl2: p=0.0894, t=2.232, C-caspase 3: p=0.2996, t=1.191, C-caspase 7: p=0.2947, t=1.205, C-caspase 9: p=0.232, t=1.408, C-caspase 12: p=0.2469, t=1.355, t-test.

(**B**) Scramble vs Scramble+SL, IRE1: p<0.0001, t=16.39, XBP1s: p=0.0079, t=4.919, CHOP: p=0.0007, t=9.627, Bcl2: p=0.0073, t=5.041, C-caspase 3: p=0.0027, t=6.63, C-caspase 7: p=0.0003, t=11.69, C-caspase 9: p=0.0005, t=10.18, C-caspase 12: p=0.0002, t=13.31; shIRE1 vs shIRE1+SL, IRE1: p=0.0594, t=2.61, XBP1s: p=0.7299, t=0.3703, CHOP: p=0.8553, t=0.1944, Bcl2: p=0.6704, t=0.4586, C-caspase 3: p=0.0725, t=2.423, C-caspase 7: p=0.1018, t=2.116, C-caspase 9: p=0.2059, t=1.509, C-caspase 12: p=0.2561, t=1.324, t-test.

Supplementary Figure S5.

(**A**) Scramble vs Scramble+SL, Bcl2: p=0.0053, t=5.521, C-caspase 3: p=0.015, t=4.088, C-caspase 7: p<0.0001, t=17.36, C-caspase 9: p=0.0011, t=8.375; Bcl2 vs Bcl2+SL, Bcl2: p=0.6444, t=0.4983,

C-caspase 3: p=0.1291, t=1.907, C-caspase 7: p=0.0769, t=2.369, C-caspase 9: p=0.6759, t=0.4502, t-test.

(**B**) Scramble vs Scramble+SL, Bcl2: p=0.041, t=2.973, C-caspase 3: p=0.0011, t=8.598, C-caspase 7: p=0.0002, t=13.03, C-caspase 9: p=0.0072, t=5.059; Bcl2 vs Bcl2+SL, Bcl2: p=0.695, t=0.415, C-caspase 3: p=0.6813, t=0.442, C-caspase 7: p=0.2126, t=1.482, C-caspase 9: p=0.7573, t=0.3309, t-test.

Supplementary Figure S6.

(**A**) GBM 3-R, DMSO vs 10 μM: p=0.0044, t=6.65, DMSO vs 20 μM: p=0.0002, t=13.11; T98 G-R, DMSO vs 10 μM: p=0.0004, t=11.21, DMSO vs 20 μM: p<0.0001, t=16.94, t-test.
